# CMRxRecon: A publicly available *k*-space dataset and benchmark to advance deep learning for cardiac MRI

**DOI:** 10.1038/s41597-024-03525-4

**Published:** 2024-06-25

**Authors:** Chengyan Wang, Jun Lyu, Shuo Wang, Chen Qin, Kunyuan Guo, Xinyu Zhang, Xiaotong Yu, Yan Li, Fanwen Wang, Jianhua Jin, Zhang Shi, Ziqiang Xu, Yapeng Tian, Sha Hua, Zhensen Chen, Meng Liu, Mengting Sun, Xutong Kuang, Kang Wang, Haoran Wang, Hao Li, Yinghua Chu, Guang Yang, Wenjia Bai, Xiahai Zhuang, He Wang, Jing Qin, Xiaobo Qu

**Affiliations:** 1 Human Phenome Institute, Fudan University, Shanghai, China; 2 Department of Psychiatry, Brigham and Women’s Hospital, Harvard Medical School, Boston, Massachusetts USA; 3 Digital Medical Research Center, School of Basic Medical Sciences, Fudan University, Shanghai, China; 4 Department of Electrical and Electronic Engineering & I-X, Imperial College London, London, UK; 5 Department of Electronic Science, Fujian Provincial Key Laboratory of Plasma and Magnetic Resonance, National Institute for Data Science in Health and Medicine, Institute of Artificial Intelligence, Xiamen University, Xiamen, China; 6 Department of Radiology, Ruijin Hospital, Shanghai Jiao Tong University School of Medicine, Shanghai, China; 7 Department of Bioengineering/Imperial-X, Imperial College London, London, UK; 8 School of Data Science, Fudan University, Shanghai, China; 9 Department of Radiology, Zhongshan Hospital, Fudan University, Shanghai, China; 10 School of Health Science and Engineering, University of Shanghai for Science and Technology, Shanghai, China; 11 Department of Computer Science, The University of Texas at Dallas, Richardson, USA; 12 Department of Cardiovascular Medicine, Ruijin Hospital Lu Wan Branch, Shanghai Jiao Tong University School of Medicine, Shanghai, China; 13 Institute of Science and Technology for Brain-Inspired Intelligence, Fudan University, Shanghai, 200433 China; 14 Simens Healthineers Ltd., Beijing, China; 15 Department of Brain Sciences, Imperial College London, London, UK; 16 Department of Computing, Imperial College London, London, UK; 17 School of Nursing, The Hong Kong Polytechnic University, Hong Kong, China

**Keywords:** Biomedical engineering, Scientific data, Diagnostic markers

## Abstract

Cardiac magnetic resonance imaging (CMR) has emerged as a valuable diagnostic tool for cardiac diseases. However, a significant drawback of CMR is its slow imaging speed, resulting in low patient throughput and compromised clinical diagnostic quality. The limited temporal resolution also causes patient discomfort and introduces artifacts in the images, further diminishing their overall quality and diagnostic value. There has been growing interest in deep learning-based CMR imaging algorithms that can reconstruct high-quality images from highly under-sampled k-space data. However, the development of deep learning methods requires large training datasets, which have so far not been made publicly available for CMR. To address this gap, we released a dataset that includes multi-contrast, multi-view, multi-slice and multi-coil CMR imaging data from 300 subjects. Imaging studies include cardiac cine and mapping sequences. The ‘CMRxRecon’ dataset contains raw k-space data and auto-calibration lines. Our aim is to facilitate the advancement of state-of-the-art CMR image reconstruction by introducing standardized evaluation criteria and making the dataset freely accessible to the research community.

## Background & Summary

Cardiac magnetic resonance (CMR) imaging has emerged as a valuable technique for diagnosing cardiovascular diseases, thanks to its superior soft tissue contrast and non-invasive nature. CMR imaging can provide anatomical, functional, and tissue characteristics information of the heart, and has been reported in many population studies^1–5^. Cine magnetic resonance imaging (MRI) is currently considered the gold standard for non-invasive evaluation of cardiac functionality. Cardiac T1 and T2 quantitative mapping are widely used in evaluating intracellular disturbances of cardiomyocytes^5^. Myocardial T1 characterization is valuable for detecting and assessing various cardiomyopathies, while T2 changes have been observed in edematous regions in patients with infarction, hemorrhage, graft rejection or myocarditis.

However, a significant drawback of CMR is its slow imaging speed, resulting in low patient throughput and compromised clinical diagnostic quality. The limited temporal resolution also causes patient discomfort and introduces artifacts in the images, further diminishing their overall quality and diagnostic value. Typically, cine is obtained using an electrocardiography (ECG)-gated segmented gradient sequence, as acquiring the full k-space data within a timeframe short enough to resolve cardiac motion accurately is not feasible. Thus, the entire k-space is segmented and read out over multiple cardiac cycles. However, for patients with impaired breath-hold capacity or cardiac arrhythmia, image degradation will occur due to the long acquisition time and motions, which can influence further diagnosis. Accelerated cine imaging effectively addresses these limitations by utilizing a reduced amount of k-space data, while still maintaining high reconstruction performance. Sufficient acceleration also enables “real-time” imaging, substantially reducing artifacts associated with respiratory motion and arrhythmia. Similar to cine imaging, accelerated T1 and T2 mapping shortens the acquisition window, leading to significant reduction of artifacts associated with respiratory motion and arrhythmia^6^. Consequently, there has been a growing interest in accelerated CMR image reconstruction from highly under-sampled k-space data.

So far, many artificial intelligence (AI)-based image reconstruction algorithms have shown great potentials in improving imaging performance^7–17^. However, deep-learning-based methods require large quantities of raw k-space data for model training. The field of CMR imaging still lacks standardized and high-quality datasets that are publicly available. In addition, due to the absence of large public datasets, there is no common gold standard on which they can be properly compared. To date, NYU Langone Health has released ‘fastMRI’ dataset, containing multi-channel brain^15^, knee^16^ and prostate^17^ MRI raw data. However, these datasets are inadequate for the 3D + 1D (time domain) scenario in cardiac imaging. To the best of our knowledge, previous available cardiac raw datasets mainly include OCMR^18^ and Harvard CMR Dataverse^19^. The former provides fully sampled as well as prospectively under-sampled cine data, while the latter released cine data with radial sampling trajectories. However, these datasets have limitations in terms of insufficient anatomical views (e.g., 2-chamber, 3-chamber), imaging contrasts (e.g., T1 and T2 mapping), and dataset size, which motivated the release of this ‘CMRxRecon’ dataset. The goal of establishing the ‘CMRxRecon’ dataset is to provide a benchmark dataset that enables the broad research community to promote advances in high quality CMR imaging.

In this paper, we describe the first release of CMR raw k-space data that includes multi-contrast, multi-view and multi- channel cardiac imaging from 300 subjects. Imaging studies include cine and mapping sequences. In addition, we released processed CMR images in The Neuroimaging Informatics Technology Initiative (NIFTI) format and the corresponding scripts of *state-of-the-art* parallel imaging.

## Methods

### Subject characteristics

The study was approved by the institutional review board of Fudan University (approval number: FE20017). The data were allowed to be made publicly available as part of the written consent process. All participants were aware of the nature of the study and agreed to make their materials publicly available in anonymized form. Inclusion criteria were defined as: 1) adults without pathologically confirmed diagnosis of cardiovascular disease, and 2) availability of an MRI examination with all imaging sequences. A total of 300 healthy volunteers (160 females and 140 males) were recruited between June 2022 and March 2023 with written informed consent. The mean age of the subjects was 26 ± 5 years.

### Image acquisition

**Fig. 1:**
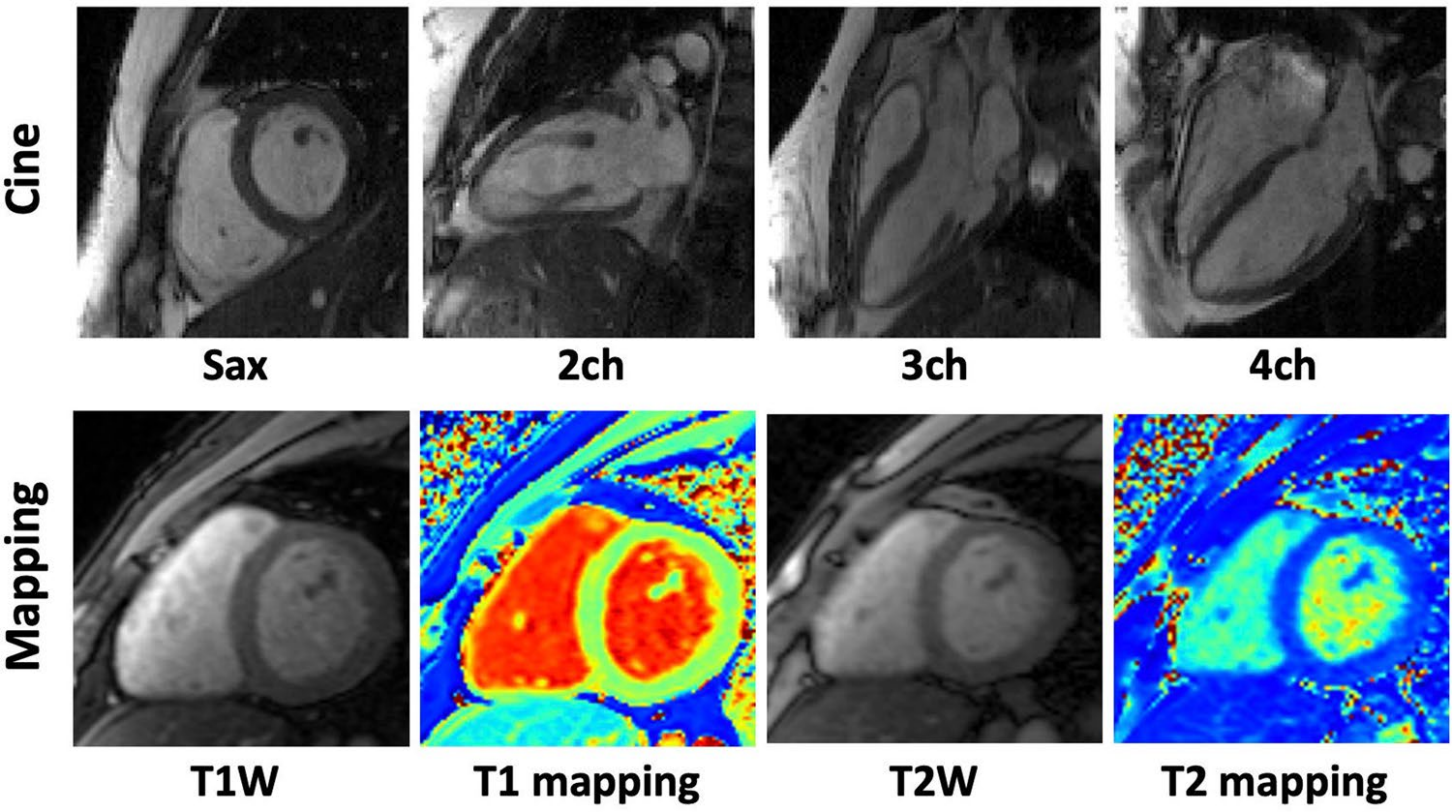
Representative CMR images of cardiac cine and mapping. Note: SAX, T1W, T2W and “ch” means short-axis, T1 weighted, T2 weighted and chamber, respectively.

Data were acquired on a 3 T scanner (MAGNETOM Vida, Siemens Healthineers, Germany), with a dedicated cardiac coil made up of 32 channels. Participants were placed in a supine position on the table before scans. Electrodes were attached and Electrocardiogram (ECG) signal were recorded during the scan. The ‘Dot’ engine was used for cardiac scout imaging. Figure 1 shows representative CMR images of cardiac cine and mapping released in the dataset.

We followed the recommendation of CMR imaging reported in the previous publication^5^. The ‘TrueFISP’ readout was used for 2D cardiac cine acquisitions. The collected images included short-axis (SAX), two-chamber (2CH), three-chamber (3CH) and four-chamber (4CH) long-axis (LAX) views. Cardiac cine was acquired through a retrospective ECG-gated segmented approach, wherein k-space is segmented in the phase encoding direction across multiple cardiac cycles. The selection of breath holds was automatically optimized according to the acquisition size, heart rate and slices. Typically, 5~14 slices were acquired for SAX view, while a single slice was acquired for each of the other views. The cardiac cycle was segmented into 12~25 phases with a temporal resolution ~50 ms according to the heart rate. Typical scan parameters were: spatial resolution of 1.5 × 1.5 mm^2^, slice thickness of 8.0 mm, repetition time (TR) of 3.6 ms, and echo-time (TE) of 1.6 ms. The parallel imaging acceleration factor was *R* = 3. Signal were acquired with breath-hold. The representative imaging parameters are summarized in Table 1.

**Table 1:** Representative scan parameters for the cardiac imaging in this dataset.

Parameter	Cine LAX	Cine SAX	T1 mapping	T2 mapping
TR (ms)	3.6	3.6	2.67	3.06
TE (ms)	1.6	1.6	1.13	1.29
Flip	48	44	35	12
Spatial resolution (mm)	1.5 × 1.5	1.5 × 1.5	1.5 × 1.5	1.5 × 1.5
Slice thickness (mm)	6.0	8.0	5.0	5.0
FOV (mm)	340 × 300	340 × 340	340 × 340	340 × 340
Number of slices	1	11	6	6
GRAPPA factor*	3.0	3.0	2.0	2.0
Breath holds	2	11	6	6

*Considering the tolerance of most individuals, a maximum breath-hold duration of 12 s was selected, leading to some acquisitions being split over two breathholds for some cases.

*The GRAPPA factor is a parameter used in parallel imaging techniques for accelerating MRI acquisition, which represents the level of under-sampling in k-space.

GRAPPA = GeneRalized Autocalibrating Partially Parallel Acquisition; FOV = field-of-view; LAX = long-axis; SAX = short-axis; TE = echo time; TR = repetition time.

T1 mapping was conducted using a modified look-locker inversion recovery (MOLLI) sequence, which acquired 9 images with different T1 weightings (using the 4-(1)-3-(1)-2 scheme, with one heart beat rest). T1 mapping was performed in SAX view only, with typical field-of-view (FOV) of 340 × 340 mm^2^, spatial resolution of 1.5 × 1.5 mm^2^, slice number of 5~6, slice thickness of 5.0 mm, TR of 2.7 ms, TE of 1.1 ms, partial Fourier of 6/8, and parallel imaging acceleration factor of *R* = 2. The inversion time varied among subjects according to the real-time heart rate. Signals were collected at the end of the diastole with ECG triggering.

T2 mapping was performed using T2-prepared (T2prep)-FLASH sequence with three T2 weightings in SAX view, with identical geometrical parameters as used in T1 mapping. Typical imaging parameters were FOV of 340 × 340 mm^2^, spatial resolution of 1.5 × 1.5 mm^2^, slice number of 5~6, slice thickness of 5.0 mm, TR of 3.0 ms, TE of 1.3 ms, T2 preparation time of 0/35/55 ms, partial Fourier of 6/8, and parallel imaging acceleration factor of *R* = 2. Signals were collected at the end of the diastole with ECG triggering.

### Image processing

**Fig. 2:**
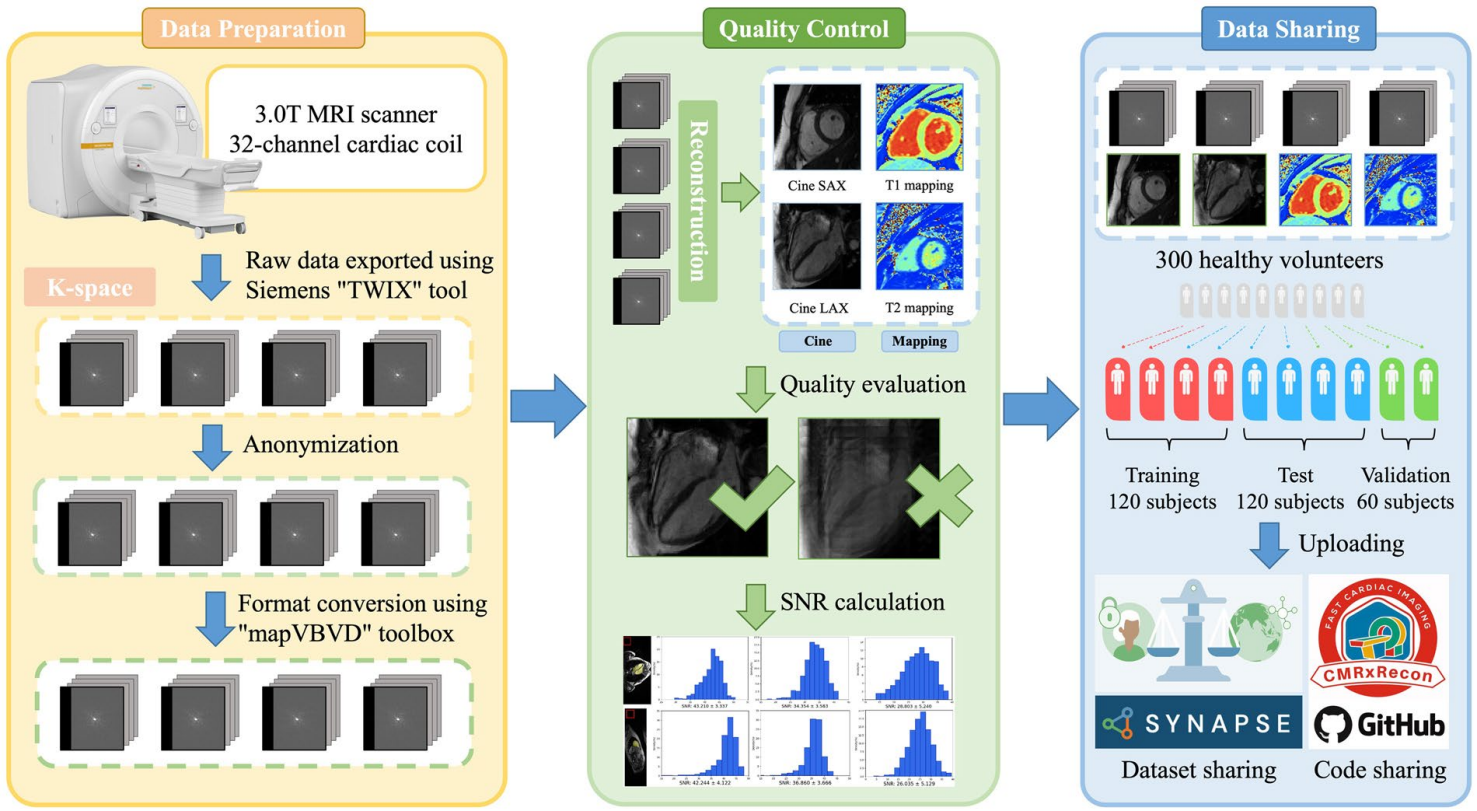
General workflow to produce the ‘CMRxRecon’ dataset. Multi-contrast, multi-view, multi-slice, multi- channel k-space data were acquired from 300 healthy volunteers using a 3.0 T MRI scanner equipped with a 32-channel cardiac coil.

The general workflow to produce the ‘CMRxRecon’ dataset is illustrated in Fig. 2. The raw data with the filename extension ‘.dat’ was exported directly from the scanner using the Siemens “TWIX” tool. The k-space data was then extracted using the “mapVBVD” toolbox (https://github.com/pehses/mapVBVD), which was written in MATLAB. The k-space data were anonymized via conversion to the raw data format. We only included imaging parameters in the raw data while removing all items related to subject identity, e.g., subject name, personal ID, hospital ID, data of exam and date of birth. Those images with poor quality were removed based on visual assessment by experienced radiologists. After these processing steps, the resulting k-space were transformed to ‘.mat’ format (MATLAB 2018a). Each k-space data we provided included 24 calibration lines, which were stored in the same file as the original data. When calculating the undersampling factor, we did not include the calibration lines. The released dataset includes 120 training data, 60 validation data and 120 test data. The training and validation datasets can be used to train reconstruction models and to determine hyperparameter values, while the test dataset is used to compare the results across different approaches. It is worth noting that there is no difference in the data processing procedures for the training, validation, and test data. Therefore, researchers can use these datasets in any combination for their studies. However, if researchers wish to compare their results with the evaluation results published for the validation and test data in the MICCAI CMRxRecon 2023 Challenge^20^, they can directly use the validation and test data provided in this study.

## Data Records

### Data description

All the data contains raw k-space data and the auto-calibration lines (ACS, 24 lines). In addition, we provided metadata of the dataset in ‘csv’ format, including FOV, acquisition matrix, slice number, slice thickness, coil number, temporal phases, TR, TE, flip angle and oversampling factor. This dataset is being made public in the Synapse repository^21^.

### Data format

The directory structure of the released dataset can be seen in Supplementary File. The CMRxRecon dataset contains two types of k-space data, i.e., raw k-space data and ACS data, both of which were complex-valued single precision multi-coil data. Detailed descriptions of the data types of cardiac cine and mapping are summarized in Table 2.

**Table 2:** Details of the data description for cardiac cine and mapping.

Coil number	File/Folder name	Dimension	Description
Cine	cine_lax_ks.mat	(kx,ky,sc,sz,t)	Complex k-space data of cine with long axis view
	cine_lax_calib.mat	(kx,ky,sc,sz,t)	Central kspace lines (ky) to be used as calibration lines with long axis view
	cine_lax_info.csv	—	Basic acquisition information for long axis view
	cine_sax_ks.mat	(kx,ky,sc,sz,t)	Complex k-space data of cine with short axis view
	cine_sax_calib.mat	(kx,ky,sc,sz,t)	Central kspace lines (ky) to be used as calibration lines with short axis view
	cine_sax_info.csv	—	Basic acquisition information for short axis view
Mapping	t1map_ks.mat	(kx,ky,sc,sz,w)	Complex k-space data of T1 mapping
	t1map_calib.mat	(kx,ky,sc,sz,w)	Central kspace lines (ky) to be used as calibration lines of T1 mapping
	t1map_info.csv	—	Basic acquisition information for T1 mapping
	t2map_ks.mat	(kx,ky,sc,sz,w)	Complex k-space data of T2 mapping
	t2map_calib.mat	(kx,ky,sc,sz,w)	Central kspace lines (ky) to be used as calibration lines of T2 mapping
	t2map_info.csv	—	Basic acquisition information for T2 mapping

Note. kx: matrix size in x-axis (k-space); ky: matrix size in y-axis (k-space); sc: coil array number; sz: slice number for short axis view, or slice group for long axis (i.e., 3ch, 2ch and 4ch views); t: time frame; w- number of weighted images.

## Technical Validation

### Evaluation on data quality

**Fig. 3:**
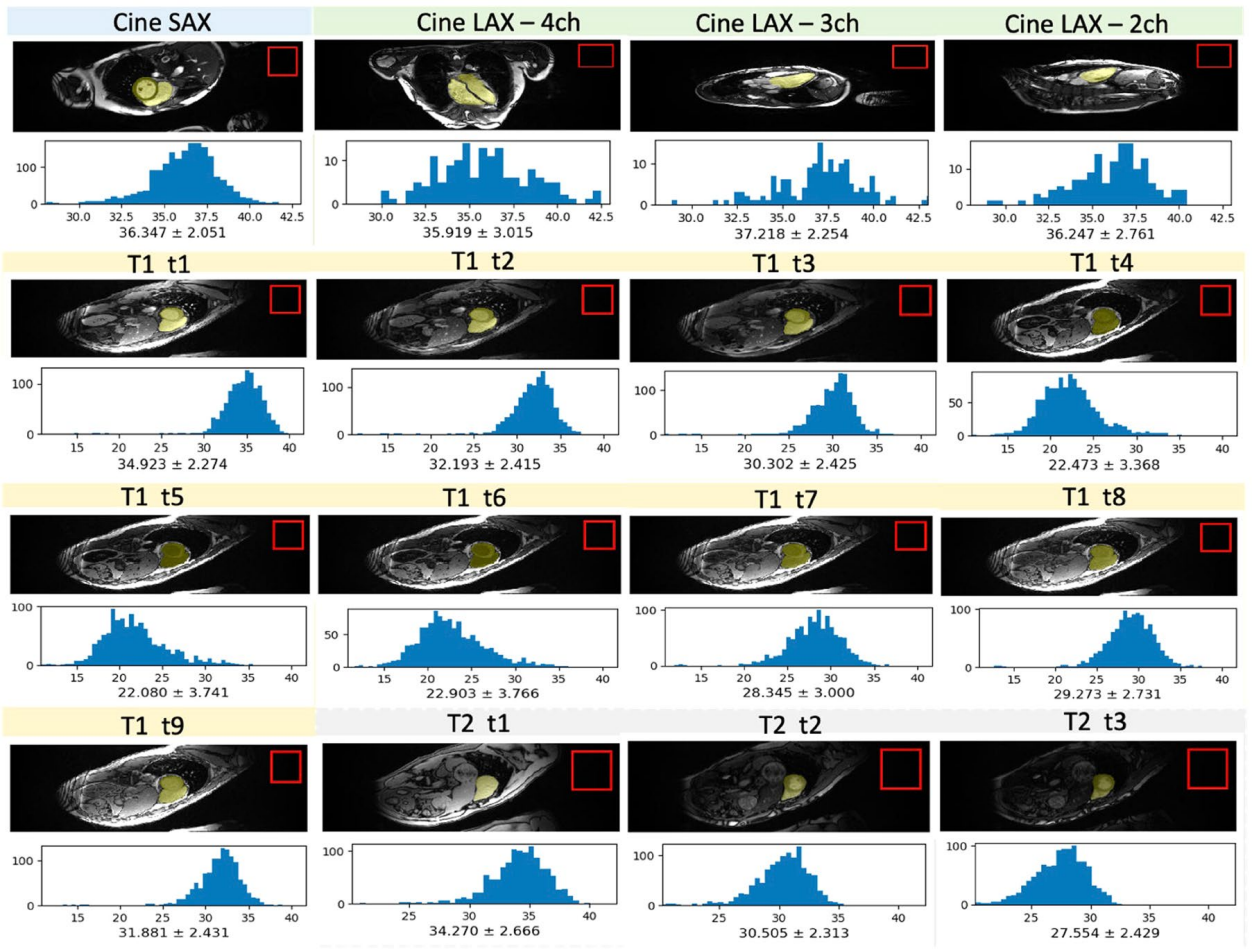
The histogram of SNR from cine, T1 mapping and T2 mapping in the reconstructed images. Different frames of T1 and T2 are shown, in which “t1-t9” following “T1” represents images at different inversion times, and “t1-t3” following “T2” represents images at different echo times. The mean and standard deviation of the SNR are labeled underneath. The noise ROI is selected from the corner (as shown in red box), while the signal ROI is selected as the whole cardiac region.

For data quality, a technician has rated the original images on a 5-point scale. We only retained images with a rating of 5 to construct the dataset. The overall signal-to-noise ratio (SNR) of the dataset is shown in Fig. 3. The SNR of the reconstructed images of both cine and mapping were measured by calculating the mean cardiac signal divided by the standard deviation of the noise. The average SNRs of all channels is 27.344 ± 9.709 for Cine SAX, 28.979 ± 8.919 for Cine LAX, 27.686 ± 8.564 for T1 mapping, and 26.212 ± 9.031 for T2 mapping, respectively. All image qualities are sufficient for cardiac region segmentation and quantitative analysis. The python scripts for image quality evaluation metrics were provided in github (https://github.com/CmrxRecon/CMRxRecon-SciData/tree/main/Evaluation).

### Benchmark reconstruction results

**Fig. 4:**
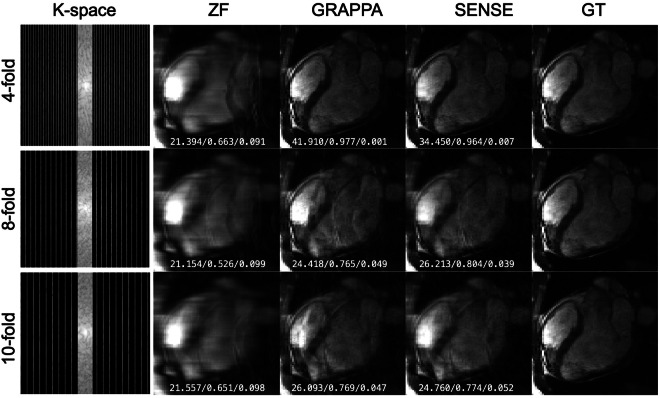
Representative Cine long-axis images in the ‘CMRxRecon’ dataset reconstructed from retrospectively undersampled k-space with benchmark algorithms. The white numbers in each subgraph represent PSNR, SSIM and NMSE, respectively.

**Fig. 5:**
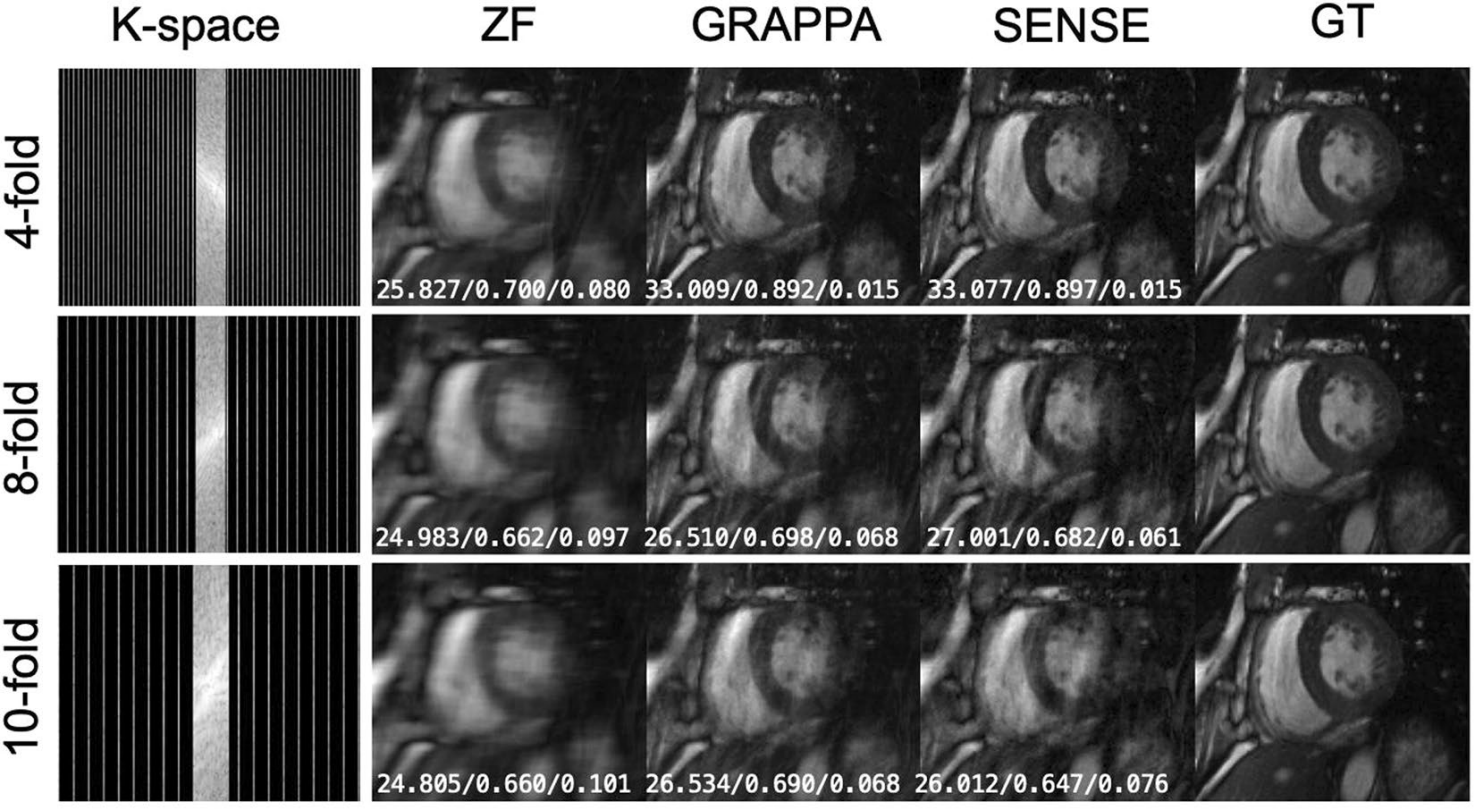
Representative Cine short-axis images in the ‘CMRxRecon’ dataset reconstructed from retrospectively undersampled k-space with benchmark algorithms. The white numbers in each subgraph represent PSNR, SSIM and NMSE, respectively.

**Fig. 6:**
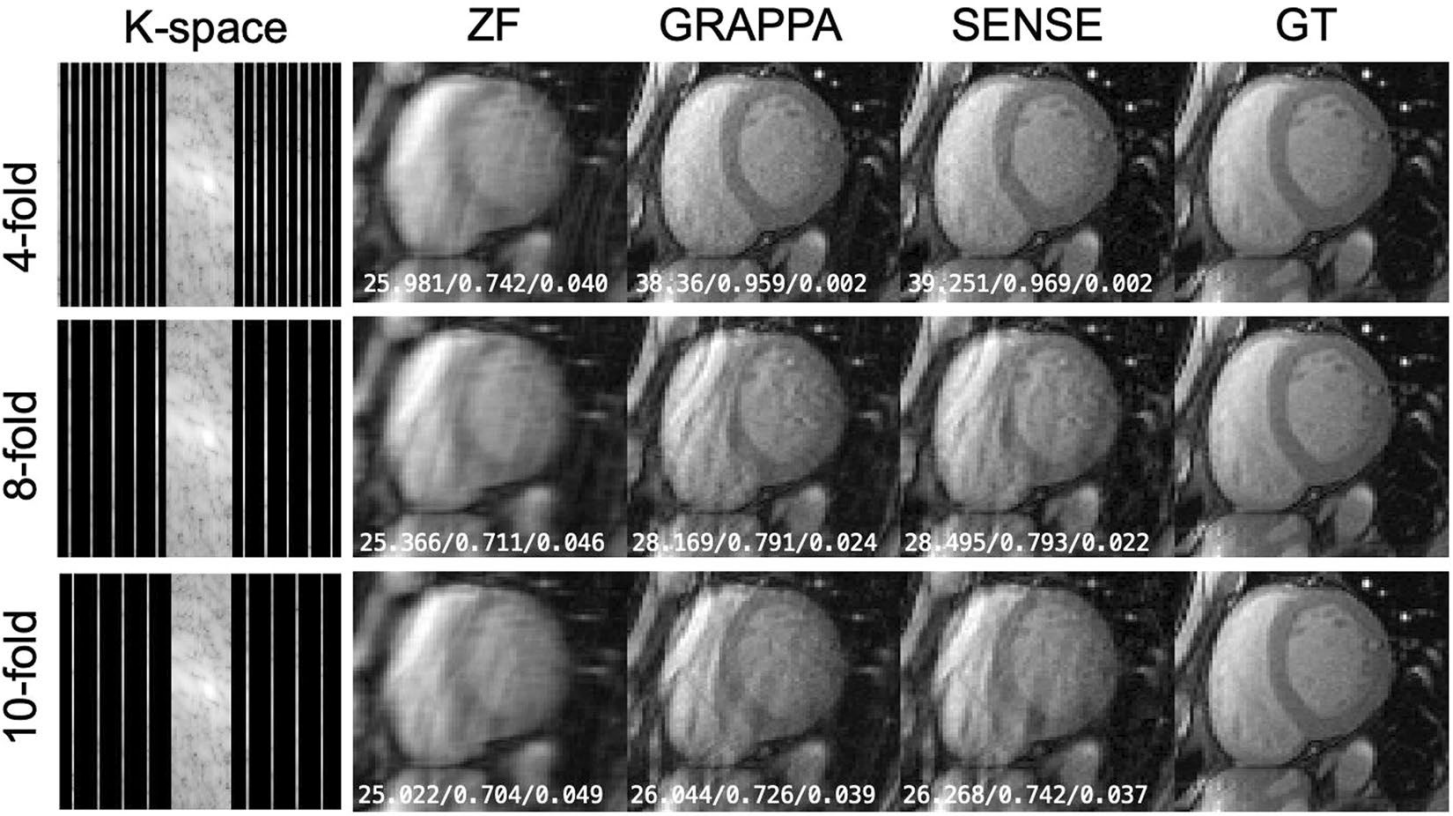
Representative T1-mapping images (1^st^ inversion time) in the ‘CMRxRecon’ dataset reconstructed from retrospectively undersampled k-space with benchmark algorithms. The white numbers in each subgraph represent PSNR, SSIM and NMSE, respectively.

**Fig. 7:**
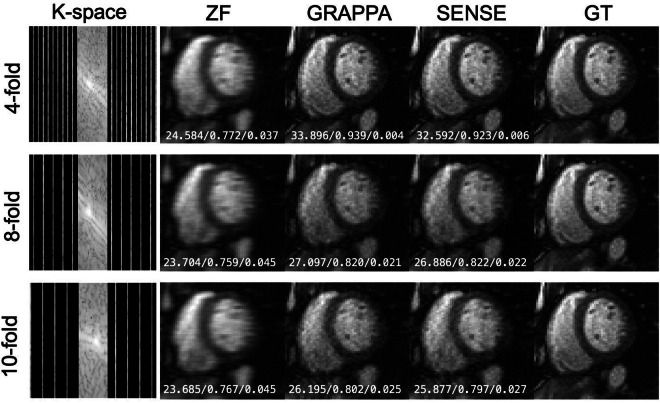
Representative T2-mapping images (3^nd^ echo) in the ‘CMRxRecon’ dataset reconstructed from retrospectively undersampled k-space with benchmark algorithms. The white numbers in each subgraph represent PSNR, SSIM and NMSE, respectively.

To further evaluate the feasibility of using the provided k-space data for undersampling image reconstruction tasks, we used the GRAPPA^22^ and ESPIRiT^23^ methods as benchmarks for examples. Representative results of the ‘CMRxRecon’ dataset with the abovementioned reconstruction algorithms are displayed in Figs. 4–7. The following criteria were used for reconstruction results assessments: normalized mean square error (NMSE), peak SNR (PSNR) and structural similarity index measure (SSIM). Quantitative assessments of the results using this benchmark algorithms are summarized in Tables 3–5. We provided scripts to reconstruct the released data using the above *state-of-the-art* algorithms in the public GitHub repository: https://github.com/CmrxRecon/CMRxRecon-SciData/tree/main/ReconCode.

**Table 3:** Quantitative assessments (PSNR) of the results in validation set using the benchmark reconstruction algorithms.

Modality	Sequence	Acceleration factor	Reconstruction method		
			ZF	GRAPPA	SENSE
Cine	Lax	4-fold	22.754 ± 1.698	33.701 ± 3.312	31.571 ± 2.453
		8-fold	22.901 ± 1.611	24.765 ± 1.357	24.840 ± 1.335
		10-fold	22.774 ± 1.629	24.687 ± 1.424	24.617 ± 1.302
	Sax	4-fold	24.426 ± 2.582	37.416 ± 3.102	36.520 ± 3.865
		8-fold	23.701 ± 2.469	27.874 ± 2.479	28.658 ± 2.835
		10-fold	23.402 ± 2.432	26.701 ± 2.412	27.289 ± 2.663
Mapping	T1 mapping	4-fold	22.996 ± 1.629	38.531 ± 3.005	38.925 ± 3.773
		8-fold	22.376 ± 1.634	27.160 ± 1.609	28.021 ± 2.275
		10-fold	22.237 ± 1.632	24.744 ± 1.428	25.883 ± 1.947
	T2 mapping	4-fold	23.886 ± 1.553	36.336 ± 2.474	34.807 ± 3.747
		8-fold	23.391 ± 1.516	27.306 ± 1.666	27.437 ± 2.775
		10-fold	23.491 ± 1.534	25.944 ± 1.438	26.190 ± 2.508

PSNR: peak signal to noise ratio; ZF: zero-filling.

**Table 4:** Quantitative assessments (SSIM) of the results in validation set using the benchmark reconstruction algorithms.

Modality	Sequence	Acceleration factor	Reconstruction method		
			ZF	GRAPPA	SENSE
Cine	Lax	4-fold	0.640 ± 0.044	0.884 ± 0.049	0.853 ± 0.056
		8-fold	0.639 ± 0.040	0.684 ± 0.041	0.674 ± 0.051
		10-fold	0.634 ± 0.039	0.687 ± 0.045	0.670 ± 0.051
	Sax	4-fold	0.718 ± 0.060	0.930 ± 0.029	0.922 ± 0.040
		8-fold	0.692 ± 0.064	0.761 ± 0.053	0.772 ± 0.060
		10-fold	0.685 ± 0.062	0.735 ± 0.055	0.741 ± 0.062
Mapping	T1 mapping	4-fold	0.657 ± 0.055	0.945 ± 0.024	0.949 ± 0.043
		8-fold	0.627 ± 0.060	0.741 ± 0.041	0.777 ± 0.058
		10-fold	0.631 ± 0.060	0.661 ± 0.045	0.707 ± 0.055
	T2 mapping	4-fold	0.768 ± 0.038	0.945 ± 0.023	0.922 ± 0.058
		8-fold	0.752 ± 0.036	0.813 ± 0.032	0.812 ± 0.069
		10-fold	0.769 ± 0.034	0.787 ± 0.032	0.787 ± 0.071

SSIM: structural similarity index measure; ZF: zero-filling.

**Table 5:** Quantitative assessments (NMSE) of the results in validation set using the benchmark reconstruction algorithms.

Modality	Sequence	Acceleration factor	Reconstruction method		
			ZF	GRAPPA	SENSE
Cine	Lax	4-fold	0.094 ± 0.033	0.017 ± 0.012	0.029 ± 0.032
		8-fold	0.096 ± 0.035	0.072 ± 0.027	0.076 ± 0.028
		10-fold	0.098 ± 0.037	0.071 ± 0.026	0.075 ± 0.030
	Sax	4-fold	0.081 ± 0.040	0.007 ± 0.006	0.012 ± 0.019
		8-fold	0.094 ± 0.045	0.043 ± 0.018	0.049 ± 0.051
		10-fold	0.099 ± 0.047	0.054 ± 0.024	0.062 ± 0.076
Mapping	T1 mapping	4-fold	0.113 ± 0.039	0.006 ± 0.004	0.016 ± 0.068
		8-fold	0.126 ± 0.043	0.054 ± 0.019	0.075 ± 0.175
		10-fold	0.130 ± 0.044	0.087 ± 0.026	0.107 ± 0.212
	T2 mapping	4-fold	0.055 ± 0.017	0.004 ± 0.003	0.026 ± 0.108
		8-fold	0.062 ± 0.020	0.028 ± 0.009	0.069 ± 0.229
		10-fold	0.062 ± 0.020	0.037 ± 0.010	0.085 ± 0.276

NMSE: normalized mean square error; ZF: zero-filling.

## Usage Notes

The dataset is public and can be downloaded from Synapse repository through this link (10.7303/syn52965326.1). Registered Synapse users are able to access the data without the need to get approved. To process the provided k-space data, it is recommended to use the tools we provided in the GitHub repository. In addition to the dataset, we also provided a platform for the evaluation of reconstruction performance (https://www.synapse.org/#!Synapse:syn51471091/wiki/622170).

### Supplementary information

Dataset 1

## Data Availability

We provide the script to facilitate the use of the released data at https://github.com/CmrxRecon/CMRxRecon-SciData. A brief description of the provided package is as follows: a) ‘ReconCode’: contains parallel imaging reconstruction code; b) ‘DemoData’: contain one example data; c) ‘Evaluation’: contains image quality evaluation code.
