# Supplementary material for: CMRxRecon: A publicly available k-space dataset and benchmark to advance deep learning for cardiac MRI
Source: Sci Data. 2024 Jun 25;11:687. doi: 10.1038/s41597-024-03525-4 (PMC11199635; doi:10.1038/s41597-024-03525-4)
Supplement: Supplementary file 1 — Dataset 1 [file 41597_2024_3525_MOESM1_ESM.pdf]

```
.
├── DemoData
│   └── P001
│       ├── cine_lax_calib.mat
│       ├── cine_lax_info.csv
│       ├── cine_lax_ks.mat
│       ├── cine_sax_calib.mat
│       ├── cine_sax_info.csv
│       ├── cine_sax_ks.mat
│       ├── t1map_calib.mat
│       ├── t1map_info.csv
│       ├── t1map_ks.mat
│       ├── t2map_calib.mat
│       ├── t2map_info.csv
│       └── t2map_ks.mat
├── TestSet
│   ├── P001
│   │   ├── cine_lax_calib.mat
│   │   ├── cine_lax_info.csv
│   │   ├── cine_lax_ks.mat
│   │   ├── cine_sax_calib.mat
│   │   ├── cine_sax_info.csv
│   │   ├── cine_sax_ks.mat
│   │   ├── t1map_calib.mat
│   │   ├── t1map_info.csv
│   │   ├── t1map_ks.mat
│   │   ├── t2map_calib.mat
│   │   ├── t2map_info.csv
│   │   └── t2map_ks.mat
│   ├── P002
│   │   ├── cine_lax_calib.mat
│   │   ├── cine_lax_info.csv
│   │   ├── cine_lax_ks.mat
│   │   ├── cine_sax_calib.mat
│   │   ├── cine_sax_info.csv
│   │   ├── cine_sax_ks.mat
│   │   ├── t1map_calib.mat
│   │   ├── t1map_info.csv
│   │   ├── t1map_ks.mat
│   │   ├── t2map_calib.mat
│   │   ├── t2map_info.csv
│   │   └── t2map_ks.mat
│   ├── P003
│   │   ├── t1map_calib.mat
│   │   ├── t1map_info.csv
│   │   ├── t1map_ks.mat
│   │   ├── t2map_calib.mat
│   │   ├── t2map_info.csv
│   │   └── t2map_ks.mat
│   ├── P004
│   │   ├── cine_lax_calib.mat
│   │   ├── cine_lax_info.csv
│   │   ├── cine_lax_ks.mat
│   │   ├── cine_sax_calib.mat
```

```
│       │       ├── cine_sax_info.csv
│       │       ├── cine_sax_ks.mat
│       │       ├── t1map_calib.mat
│       │       ├── t1map_info.csv
│       │       ├── t1map_ks.mat
│       │       ├── t2map_calib.mat
│       │       ├── t2map_info.csv
│       │       └── t2map_ks.mat
│       ├── P005
│       │       ├── cine_lax_calib.mat
│       │       ├── cine_lax_info.csv
│       │       ├── cine_lax_ks.mat
│       │       ├── cine_sax_calib.mat
│       │       ├── cine_sax_info.csv
│       │       ├── cine_sax_ks.mat
│       │       ├── t1map_calib.mat
│       │       ├── t1map_info.csv
│       │       ├── t1map_ks.mat
│       │       ├── t2map_calib.mat
│       │       ├── t2map_info.csv
│       │       └── t2map_ks.mat
│       ├── P006
│       │       ├── cine_lax_calib.mat
│       │       ├── cine_lax_info.csv
│       │       ├── cine_lax_ks.mat
│       │       ├── cine_sax_calib.mat
│       │       ├── cine_sax_info.csv
│       │       ├── cine_sax_ks.mat
│       │       ├── t1map_calib.mat
│       │       ├── t1map_info.csv
│       │       ├── t1map_ks.mat
│       │       ├── t2map_calib.mat
│       │       ├── t2map_info.csv
│       │       └── t2map_ks.mat
│       ├── P007
│       │       ├── t1map_calib.mat
│       │       ├── t1map_info.csv
│       │       ├── t1map_ks.mat
│       │       ├── t2map_calib.mat
│       │       ├── t2map_info.csv
│       │       └── t2map_ks.mat
│       ├── P008
│       │       ├── t1map_calib.mat
│       │       ├── t1map_info.csv
│       │       ├── t1map_ks.mat
│       │       ├── t2map_calib.mat
│       │       ├── t2map_info.csv
│       │       └── t2map_ks.mat
│       ├── P009
│       │       ├── t1map_calib.mat
│       │       ├── t1map_info.csv
│       │       ├── t1map_ks.mat
│       │       ├── t2map_calib.mat
│       │       ├── t2map_info.csv
```

```
│       │   └── t2map_ks.mat
│       ├── P010
│       │   ├── t1map_calib.mat
│       │   ├── t1map_info.csv
│       │   ├── t1map_ks.mat
│       │   ├── t2map_calib.mat
│       │   ├── t2map_info.csv
│       │   └── t2map_ks.mat
│       ├── P011
│       │   ├── cine_lax_calib.mat
│       │   ├── cine_lax_info.csv
│       │   ├── cine_lax_ks.mat
│       │   ├── t1map_calib.mat
│       │   ├── t1map_info.csv
│       │   ├── t1map_ks.mat
│       │   ├── t2map_calib.mat
│       │   ├── t2map_info.csv
│       │   └── t2map_ks.mat
│       ├── P012
│       │   ├── cine_lax_calib.mat
│       │   ├── cine_lax_info.csv
│       │   ├── cine_lax_ks.mat
│       │   ├── cine_sax_calib.mat
│       │   ├── cine_sax_info.csv
│       │   ├── cine_sax_ks.mat
│       │   ├── t1map_calib.mat
│       │   ├── t1map_info.csv
│       │   ├── t1map_ks.mat
│       │   ├── t2map_calib.mat
│       │   ├── t2map_info.csv
│       │   └── t2map_ks.mat
│       ├── P013
│       │   ├── cine_lax_calib.mat
│       │   ├── cine_lax_info.csv
│       │   ├── cine_lax_ks.mat
│       │   ├── cine_sax_calib.mat
│       │   ├── cine_sax_info.csv
│       │   ├── cine_sax_ks.mat
│       │   ├── t1map_calib.mat
│       │   ├── t1map_info.csv
│       │   ├── t1map_ks.mat
│       │   ├── t2map_calib.mat
│       │   ├── t2map_info.csv
│       │   └── t2map_ks.mat
│       ├── P014
│       │   ├── t1map_calib.mat
│       │   ├── t1map_info.csv
│       │   ├── t1map_ks.mat
│       │   ├── t2map_calib.mat
│       │   ├── t2map_info.csv
│       │   └── t2map_ks.mat
│       ├── P015
│       │   ├── cine_sax_calib.mat
│       │   ├── cine_sax_info.csv
```

```
│       │       ├── cine_sax_ks.mat
│       │       ├── t1map_calib.mat
│       │       ├── t1map_info.csv
│       │       ├── t1map_ks.mat
│       │       ├── t2map_calib.mat
│       │       ├── t2map_info.csv
│       │       └── t2map_ks.mat
│       ├── P016
│       │       ├── t1map_calib.mat
│       │       ├── t1map_info.csv
│       │       ├── t1map_ks.mat
│       │       ├── t2map_calib.mat
│       │       ├── t2map_info.csv
│       │       └── t2map_ks.mat
│       ├── P017
│       │       ├── t1map_calib.mat
│       │       ├── t1map_info.csv
│       │       ├── t1map_ks.mat
│       │       ├── t2map_calib.mat
│       │       ├── t2map_info.csv
│       │       └── t2map_ks.mat
│       ├── P018
│       │       ├── cine_lax_calib.mat
│       │       ├── cine_lax_info.csv
│       │       ├── cine_lax_ks.mat
│       │       ├── cine_sax_calib.mat
│       │       ├── cine_sax_info.csv
│       │       ├── cine_sax_ks.mat
│       │       ├── t1map_calib.mat
│       │       ├── t1map_info.csv
│       │       ├── t1map_ks.mat
│       │       ├── t2map_calib.mat
│       │       ├── t2map_info.csv
│       │       └── t2map_ks.mat
│       ├── P019
│       │       ├── cine_lax_calib.mat
│       │       ├── cine_lax_info.csv
│       │       ├── cine_lax_ks.mat
│       │       ├── cine_sax_calib.mat
│       │       ├── cine_sax_info.csv
│       │       ├── cine_sax_ks.mat
│       │       ├── t1map_calib.mat
│       │       ├── t1map_info.csv
│       │       ├── t1map_ks.mat
│       │       ├── t2map_calib.mat
│       │       ├── t2map_info.csv
│       │       └── t2map_ks.mat
│       ├── P020
│       │       ├── cine_lax_calib.mat
│       │       ├── cine_lax_info.csv
│       │       ├── cine_lax_ks.mat
│       │       ├── cine_sax_calib.mat
│       │       ├── cine_sax_info.csv
│       │       ├── cine_sax_ks.mat
```

```
│       │   ├── t1map_calib.mat
│       │   ├── t1map_info.csv
│       │   ├── t1map_ks.mat
│       │   ├── t2map_calib.mat
│       │   ├── t2map_info.csv
│       │   └── t2map_ks.mat
│       ├── P021
│       │   ├── cine_lax_calib.mat
│       │   ├── cine_lax_info.csv
│       │   ├── cine_lax_ks.mat
│       │   ├── cine_sax_calib.mat
│       │   ├── cine_sax_info.csv
│       │   ├── cine_sax_ks.mat
│       │   ├── t1map_calib.mat
│       │   ├── t1map_info.csv
│       │   ├── t1map_ks.mat
│       │   ├── t2map_calib.mat
│       │   ├── t2map_info.csv
│       │   └── t2map_ks.mat
│       ├── P022
│       │   ├── cine_lax_calib.mat
│       │   ├── cine_lax_info.csv
│       │   ├── cine_lax_ks.mat
│       │   ├── cine_sax_calib.mat
│       │   ├── cine_sax_info.csv
│       │   ├── cine_sax_ks.mat
│       │   ├── t1map_calib.mat
│       │   ├── t1map_info.csv
│       │   ├── t1map_ks.mat
│       │   ├── t2map_calib.mat
│       │   ├── t2map_info.csv
│       │   └── t2map_ks.mat
│       ├── P023
│       │   ├── t1map_calib.mat
│       │   ├── t1map_info.csv
│       │   ├── t1map_ks.mat
│       │   ├── t2map_calib.mat
│       │   ├── t2map_info.csv
│       │   └── t2map_ks.mat
│       ├── P024
│       │   ├── t1map_calib.mat
│       │   ├── t1map_info.csv
│       │   ├── t1map_ks.mat
│       │   ├── t2map_calib.mat
│       │   ├── t2map_info.csv
│       │   └── t2map_ks.mat
│       ├── P025
│       │   ├── cine_lax_calib.mat
│       │   ├── cine_lax_info.csv
│       │   ├── cine_lax_ks.mat
│       │   ├── cine_sax_calib.mat
│       │   ├── cine_sax_info.csv
│       │   ├── cine_sax_ks.mat
│       │   ├── t1map_calib.mat
```

```
│   │   ├── t1map_info.csv
│   │   ├── t1map_ks.mat
│   │   ├── t2map_calib.mat
│   │   ├── t2map_info.csv
│   │   └── t2map_ks.mat
│   ├── P026
│   │   ├── cine_lax_calib.mat
│   │   ├── cine_lax_info.csv
│   │   ├── cine_lax_ks.mat
│   │   ├── cine_sax_calib.mat
│   │   ├── cine_sax_info.csv
│   │   ├── cine_sax_ks.mat
│   │   ├── t1map_calib.mat
│   │   ├── t1map_info.csv
│   │   ├── t1map_ks.mat
│   │   ├── t2map_calib.mat
│   │   ├── t2map_info.csv
│   │   └── t2map_ks.mat
│   ├── P027
│   │   ├── cine_lax_calib.mat
│   │   ├── cine_lax_info.csv
│   │   ├── cine_lax_ks.mat
│   │   ├── cine_sax_calib.mat
│   │   ├── cine_sax_info.csv
│   │   ├── cine_sax_ks.mat
│   │   ├── t1map_calib.mat
│   │   ├── t1map_info.csv
│   │   ├── t1map_ks.mat
│   │   ├── t2map_calib.mat
│   │   ├── t2map_info.csv
│   │   └── t2map_ks.mat
│   ├── P028
│   │   ├── cine_sax_calib.mat
│   │   ├── cine_sax_info.csv
│   │   ├── cine_sax_ks.mat
│   │   ├── t1map_calib.mat
│   │   ├── t1map_info.csv
│   │   ├── t1map_ks.mat
│   │   ├── t2map_calib.mat
│   │   ├── t2map_info.csv
│   │   └── t2map_ks.mat
│   ├── P029
│   │   ├── cine_lax_calib.mat
│   │   ├── cine_lax_info.csv
│   │   ├── cine_lax_ks.mat
│   │   ├── cine_sax_calib.mat
│   │   ├── cine_sax_info.csv
│   │   ├── cine_sax_ks.mat
│   │   ├── t1map_calib.mat
│   │   ├── t1map_info.csv
│   │   ├── t1map_ks.mat
│   │   ├── t2map_calib.mat
│   │   ├── t2map_info.csv
│   │   └── t2map_ks.mat
│   │
```

```
│       ├── P030
│       │   ├── cine_lax_calib.mat
│       │   ├── cine_lax_info.csv
│       │   ├── cine_lax_ks.mat
│       │   ├── cine_sax_calib.mat
│       │   ├── cine_sax_info.csv
│       │   ├── cine_sax_ks.mat
│       │   ├── t1map_calib.mat
│       │   ├── t1map_info.csv
│       │   ├── t1map_ks.mat
│       │   ├── t2map_calib.mat
│       │   ├── t2map_info.csv
│       │   └── t2map_ks.mat
│       ├── P031
│       │   ├── cine_lax_calib.mat
│       │   ├── cine_lax_info.csv
│       │   ├── cine_lax_ks.mat
│       │   ├── cine_sax_calib.mat
│       │   ├── cine_sax_info.csv
│       │   ├── cine_sax_ks.mat
│       │   ├── t1map_calib.mat
│       │   ├── t1map_info.csv
│       │   ├── t1map_ks.mat
│       │   ├── t2map_calib.mat
│       │   ├── t2map_info.csv
│       │   └── t2map_ks.mat
│       ├── P032
│       │   ├── cine_lax_calib.mat
│       │   ├── cine_lax_info.csv
│       │   ├── cine_lax_ks.mat
│       │   ├── cine_sax_calib.mat
│       │   ├── cine_sax_info.csv
│       │   ├── cine_sax_ks.mat
│       │   ├── t1map_calib.mat
│       │   ├── t1map_info.csv
│       │   ├── t1map_ks.mat
│       │   ├── t2map_calib.mat
│       │   ├── t2map_info.csv
│       │   └── t2map_ks.mat
│       ├── P033
│       │   ├── cine_lax_calib.mat
│       │   ├── cine_lax_info.csv
│       │   ├── cine_lax_ks.mat
│       │   ├── cine_sax_calib.mat
│       │   ├── cine_sax_info.csv
│       │   ├── cine_sax_ks.mat
│       │   ├── t1map_calib.mat
│       │   ├── t1map_info.csv
│       │   ├── t1map_ks.mat
│       │   ├── t2map_calib.mat
│       │   ├── t2map_info.csv
│       │   └── t2map_ks.mat
│       ├── P034
│       │   ├── cine_lax_calib.mat
```

```
│       │       ├── cine_lax_info.csv
│       │       ├── cine_lax_ks.mat
│       │       ├── cine_sax_calib.mat
│       │       ├── cine_sax_info.csv
│       │       ├── cine_sax_ks.mat
│       │       ├── t1map_calib.mat
│       │       ├── t1map_info.csv
│       │       ├── t1map_ks.mat
│       │       ├── t2map_calib.mat
│       │       ├── t2map_info.csv
│       │       └── t2map_ks.mat
│       ├── P035
│       │       ├── cine_lax_calib.mat
│       │       ├── cine_lax_info.csv
│       │       ├── cine_lax_ks.mat
│       │       ├── cine_sax_calib.mat
│       │       ├── cine_sax_info.csv
│       │       ├── cine_sax_ks.mat
│       │       ├── t1map_calib.mat
│       │       ├── t1map_info.csv
│       │       ├── t1map_ks.mat
│       │       ├── t2map_calib.mat
│       │       ├── t2map_info.csv
│       │       └── t2map_ks.mat
│       ├── P036
│       │       ├── cine_lax_calib.mat
│       │       ├── cine_lax_info.csv
│       │       ├── cine_lax_ks.mat
│       │       ├── cine_sax_calib.mat
│       │       ├── cine_sax_info.csv
│       │       ├── cine_sax_ks.mat
│       │       ├── t1map_calib.mat
│       │       ├── t1map_info.csv
│       │       ├── t1map_ks.mat
│       │       ├── t2map_calib.mat
│       │       ├── t2map_info.csv
│       │       └── t2map_ks.mat
│       ├── P037
│       │       ├── t1map_calib.mat
│       │       ├── t1map_info.csv
│       │       ├── t1map_ks.mat
│       │       ├── t2map_calib.mat
│       │       ├── t2map_info.csv
│       │       └── t2map_ks.mat
│       ├── P038
│       │       ├── cine_lax_calib.mat
│       │       ├── cine_lax_info.csv
│       │       ├── cine_lax_ks.mat
│       │       ├── cine_sax_calib.mat
│       │       ├── cine_sax_info.csv
│       │       ├── cine_sax_ks.mat
│       │       ├── t1map_calib.mat
│       │       ├── t1map_info.csv
│       │       ├── t1map_ks.mat
```

```
│       │       ├── t2map_calib.mat
│       │       ├── t2map_info.csv
│       │       └── t2map_ks.mat
│       ├── P039
│       │       ├── t1map_calib.mat
│       │       ├── t1map_info.csv
│       │       ├── t1map_ks.mat
│       │       ├── t2map_calib.mat
│       │       ├── t2map_info.csv
│       │       └── t2map_ks.mat
│       ├── P040
│       │       ├── cine_lax_calib.mat
│       │       ├── cine_lax_info.csv
│       │       ├── cine_lax_ks.mat
│       │       ├── cine_sax_calib.mat
│       │       ├── cine_sax_info.csv
│       │       ├── cine_sax_ks.mat
│       │       ├── t1map_calib.mat
│       │       ├── t1map_info.csv
│       │       ├── t1map_ks.mat
│       │       ├── t2map_calib.mat
│       │       ├── t2map_info.csv
│       │       └── t2map_ks.mat
│       ├── P041
│       │       ├── cine_lax_calib.mat
│       │       ├── cine_lax_info.csv
│       │       ├── cine_lax_ks.mat
│       │       ├── cine_sax_calib.mat
│       │       ├── cine_sax_info.csv
│       │       ├── cine_sax_ks.mat
│       │       ├── t1map_calib.mat
│       │       ├── t1map_info.csv
│       │       ├── t1map_ks.mat
│       │       ├── t2map_calib.mat
│       │       ├── t2map_info.csv
│       │       └── t2map_ks.mat
│       ├── P042
│       │       ├── cine_lax_calib.mat
│       │       ├── cine_lax_info.csv
│       │       ├── cine_lax_ks.mat
│       │       ├── cine_sax_calib.mat
│       │       ├── cine_sax_info.csv
│       │       └── cine_sax_ks.mat
│       ├── P043
│       │       ├── cine_lax_calib.mat
│       │       ├── cine_lax_info.csv
│       │       ├── cine_lax_ks.mat
│       │       ├── cine_sax_calib.mat
│       │       ├── cine_sax_info.csv
│       │       ├── cine_sax_ks.mat
│       │       ├── t1map_calib.mat
│       │       ├── t1map_info.csv
│       │       ├── t1map_ks.mat
│       │       ├── t2map_calib.mat
```

```
│       │    ├── t2map_info.csv
│       │    └── t2map_ks.mat
│       ├── P044
│       │    ├── cine_lax_calib.mat
│       │    ├── cine_lax_info.csv
│       │    ├── cine_lax_ks.mat
│       │    ├── cine_sax_calib.mat
│       │    ├── cine_sax_info.csv
│       │    ├── cine_sax_ks.mat
│       │    ├── t1map_calib.mat
│       │    ├── t1map_info.csv
│       │    ├── t1map_ks.mat
│       │    ├── t2map_calib.mat
│       │    ├── t2map_info.csv
│       │    └── t2map_ks.mat
│       ├── P045
│       │    ├── cine_sax_calib.mat
│       │    ├── cine_sax_info.csv
│       │    ├── cine_sax_ks.mat
│       │    ├── t1map_calib.mat
│       │    ├── t1map_info.csv
│       │    ├── t1map_ks.mat
│       │    ├── t2map_calib.mat
│       │    ├── t2map_info.csv
│       │    └── t2map_ks.mat
│       ├── P046
│       │    ├── cine_lax_calib.mat
│       │    ├── cine_lax_info.csv
│       │    ├── cine_lax_ks.mat
│       │    ├── cine_sax_calib.mat
│       │    ├── cine_sax_info.csv
│       │    ├── cine_sax_ks.mat
│       │    ├── t1map_calib.mat
│       │    ├── t1map_info.csv
│       │    ├── t1map_ks.mat
│       │    ├── t2map_calib.mat
│       │    ├── t2map_info.csv
│       │    └── t2map_ks.mat
│       ├── P047
│       │    ├── cine_lax_calib.mat
│       │    ├── cine_lax_info.csv
│       │    ├── cine_lax_ks.mat
│       │    ├── cine_sax_calib.mat
│       │    ├── cine_sax_info.csv
│       │    ├── cine_sax_ks.mat
│       │    ├── t1map_calib.mat
│       │    ├── t1map_info.csv
│       │    ├── t1map_ks.mat
│       │    ├── t2map_calib.mat
│       │    ├── t2map_info.csv
│       │    └── t2map_ks.mat
│       ├── P048
│       │    ├── cine_lax_calib.mat
│       │    ├── cine_lax_info.csv
```

```
│       │       ├── cine_lax_ks.mat
│       │       ├── cine_sax_calib.mat
│       │       ├── cine_sax_info.csv
│       │       ├── cine_sax_ks.mat
│       │       ├── t1map_calib.mat
│       │       ├── t1map_info.csv
│       │       ├── t1map_ks.mat
│       │       ├── t2map_calib.mat
│       │       ├── t2map_info.csv
│       │       └── t2map_ks.mat
│       ├── P049
│       │       ├── cine_lax_calib.mat
│       │       ├── cine_lax_info.csv
│       │       ├── cine_lax_ks.mat
│       │       ├── cine_sax_calib.mat
│       │       ├── cine_sax_info.csv
│       │       ├── cine_sax_ks.mat
│       │       ├── t1map_calib.mat
│       │       ├── t1map_info.csv
│       │       ├── t1map_ks.mat
│       │       ├── t2map_calib.mat
│       │       ├── t2map_info.csv
│       │       └── t2map_ks.mat
│       ├── P050
│       │       ├── cine_lax_calib.mat
│       │       ├── cine_lax_info.csv
│       │       ├── cine_lax_ks.mat
│       │       ├── cine_sax_calib.mat
│       │       ├── cine_sax_info.csv
│       │       ├── cine_sax_ks.mat
│       │       ├── t1map_calib.mat
│       │       ├── t1map_info.csv
│       │       ├── t1map_ks.mat
│       │       ├── t2map_calib.mat
│       │       ├── t2map_info.csv
│       │       └── t2map_ks.mat
│       ├── P051
│       │       ├── cine_lax_calib.mat
│       │       ├── cine_lax_info.csv
│       │       ├── cine_lax_ks.mat
│       │       ├── cine_sax_calib.mat
│       │       ├── cine_sax_info.csv
│       │       ├── cine_sax_ks.mat
│       │       ├── t1map_calib.mat
│       │       ├── t1map_info.csv
│       │       ├── t1map_ks.mat
│       │       ├── t2map_calib.mat
│       │       ├── t2map_info.csv
│       │       └── t2map_ks.mat
│       ├── P052
│       │       ├── cine_sax_calib.mat
│       │       ├── cine_sax_info.csv
│       │       ├── cine_sax_ks.mat
│       │       ├── t1map_calib.mat
```

```
│       │   ├── t1map_info.csv
│       │   ├── t1map_ks.mat
│       │   ├── t2map_calib.mat
│       │   ├── t2map_info.csv
│       │   └── t2map_ks.mat
│       ├── P053
│       │   ├── cine_lax_calib.mat
│       │   ├── cine_lax_info.csv
│       │   ├── cine_lax_ks.mat
│       │   ├── cine_sax_calib.mat
│       │   ├── cine_sax_info.csv
│       │   ├── cine_sax_ks.mat
│       │   ├── t1map_calib.mat
│       │   ├── t1map_info.csv
│       │   ├── t1map_ks.mat
│       │   ├── t2map_calib.mat
│       │   ├── t2map_info.csv
│       │   └── t2map_ks.mat
│       ├── P054
│       │   ├── cine_lax_calib.mat
│       │   ├── cine_lax_info.csv
│       │   ├── cine_lax_ks.mat
│       │   ├── cine_sax_calib.mat
│       │   ├── cine_sax_info.csv
│       │   ├── cine_sax_ks.mat
│       │   ├── t1map_calib.mat
│       │   ├── t1map_info.csv
│       │   ├── t1map_ks.mat
│       │   ├── t2map_calib.mat
│       │   ├── t2map_info.csv
│       │   └── t2map_ks.mat
│       ├── P055
│       │   ├── cine_sax_calib.mat
│       │   ├── cine_sax_info.csv
│       │   ├── cine_sax_ks.mat
│       │   ├── t1map_calib.mat
│       │   ├── t1map_info.csv
│       │   ├── t1map_ks.mat
│       │   ├── t2map_calib.mat
│       │   ├── t2map_info.csv
│       │   └── t2map_ks.mat
│       ├── P056
│       │   ├── cine_lax_calib.mat
│       │   ├── cine_lax_info.csv
│       │   ├── cine_lax_ks.mat
│       │   ├── cine_sax_calib.mat
│       │   ├── cine_sax_info.csv
│       │   ├── cine_sax_ks.mat
│       │   ├── t1map_calib.mat
│       │   ├── t1map_info.csv
│       │   ├── t1map_ks.mat
│       │   ├── t2map_calib.mat
│       │   ├── t2map_info.csv
│       │   └── t2map_ks.mat
```

```
│       ├── P057
│       │   ├── t1map_calib.mat
│       │   ├── t1map_info.csv
│       │   ├── t1map_ks.mat
│       │   ├── t2map_calib.mat
│       │   ├── t2map_info.csv
│       │   └── t2map_ks.mat
│       ├── P058
│       │   ├── t1map_calib.mat
│       │   ├── t1map_info.csv
│       │   ├── t1map_ks.mat
│       │   ├── t2map_calib.mat
│       │   ├── t2map_info.csv
│       │   └── t2map_ks.mat
│       ├── P059
│       │   ├── cine_lax_calib.mat
│       │   ├── cine_lax_info.csv
│       │   ├── cine_lax_ks.mat
│       │   ├── cine_sax_calib.mat
│       │   ├── cine_sax_info.csv
│       │   ├── cine_sax_ks.mat
│       │   ├── t1map_calib.mat
│       │   ├── t1map_info.csv
│       │   ├── t1map_ks.mat
│       │   ├── t2map_calib.mat
│       │   ├── t2map_info.csv
│       │   └── t2map_ks.mat
│       ├── P060
│       │   ├── cine_lax_calib.mat
│       │   ├── cine_lax_info.csv
│       │   ├── cine_lax_ks.mat
│       │   ├── cine_sax_calib.mat
│       │   ├── cine_sax_info.csv
│       │   ├── cine_sax_ks.mat
│       │   ├── t1map_calib.mat
│       │   ├── t1map_info.csv
│       │   ├── t1map_ks.mat
│       │   ├── t2map_calib.mat
│       │   ├── t2map_info.csv
│       │   └── t2map_ks.mat
│       ├── P061
│       │   ├── cine_lax_calib.mat
│       │   ├── cine_lax_info.csv
│       │   ├── cine_lax_ks.mat
│       │   ├── cine_sax_calib.mat
│       │   ├── cine_sax_info.csv
│       │   ├── cine_sax_ks.mat
│       │   ├── t1map_calib.mat
│       │   ├── t1map_info.csv
│       │   ├── t1map_ks.mat
│       │   ├── t2map_calib.mat
│       │   ├── t2map_info.csv
│       │   └── t2map_ks.mat
│       ├── P062
```

```
│   │   ├── t1map_calib.mat
│   │   ├── t1map_info.csv
│   │   ├── t1map_ks.mat
│   │   ├── t2map_calib.mat
│   │   ├── t2map_info.csv
│   │   └── t2map_ks.mat
│   ├── P063
│   │   ├── cine_lax_calib.mat
│   │   ├── cine_lax_info.csv
│   │   ├── cine_lax_ks.mat
│   │   ├── cine_sax_calib.mat
│   │   ├── cine_sax_info.csv
│   │   ├── cine_sax_ks.mat
│   │   ├── t1map_calib.mat
│   │   ├── t1map_info.csv
│   │   ├── t1map_ks.mat
│   │   ├── t2map_calib.mat
│   │   ├── t2map_info.csv
│   │   └── t2map_ks.mat
│   ├── P064
│   │   ├── t1map_calib.mat
│   │   ├── t1map_info.csv
│   │   ├── t1map_ks.mat
│   │   ├── t2map_calib.mat
│   │   ├── t2map_info.csv
│   │   └── t2map_ks.mat
│   ├── P065
│   │   ├── cine_lax_calib.mat
│   │   ├── cine_lax_info.csv
│   │   ├── cine_lax_ks.mat
│   │   ├── cine_sax_calib.mat
│   │   ├── cine_sax_info.csv
│   │   ├── cine_sax_ks.mat
│   │   ├── t1map_calib.mat
│   │   ├── t1map_info.csv
│   │   ├── t1map_ks.mat
│   │   ├── t2map_calib.mat
│   │   ├── t2map_info.csv
│   │   └── t2map_ks.mat
│   ├── P066
│   │   ├── cine_lax_calib.mat
│   │   ├── cine_lax_info.csv
│   │   ├── cine_lax_ks.mat
│   │   ├── cine_sax_calib.mat
│   │   ├── cine_sax_info.csv
│   │   ├── cine_sax_ks.mat
│   │   ├── t1map_calib.mat
│   │   ├── t1map_info.csv
│   │   ├── t1map_ks.mat
│   │   ├── t2map_calib.mat
│   │   ├── t2map_info.csv
│   │   └── t2map_ks.mat
│   ├── P067
│   │   ├── cine_lax_calib.mat
```

```
│   │   ├── cine_lax_info.csv
│   │   ├── cine_lax_ks.mat
│   │   ├── cine_sax_calib.mat
│   │   ├── cine_sax_info.csv
│   │   ├── cine_sax_ks.mat
│   │   ├── t1map_calib.mat
│   │   ├── t1map_info.csv
│   │   ├── t1map_ks.mat
│   │   ├── t2map_calib.mat
│   │   ├── t2map_info.csv
│   │   └── t2map_ks.mat
│   ├── P068
│   │   ├── cine_lax_calib.mat
│   │   ├── cine_lax_info.csv
│   │   ├── cine_lax_ks.mat
│   │   ├── cine_sax_calib.mat
│   │   ├── cine_sax_info.csv
│   │   ├── cine_sax_ks.mat
│   │   ├── t1map_calib.mat
│   │   ├── t1map_info.csv
│   │   ├── t1map_ks.mat
│   │   ├── t2map_calib.mat
│   │   ├── t2map_info.csv
│   │   └── t2map_ks.mat
│   ├── P069
│   │   ├── cine_lax_calib.mat
│   │   ├── cine_lax_info.csv
│   │   ├── cine_lax_ks.mat
│   │   ├── cine_sax_calib.mat
│   │   ├── cine_sax_info.csv
│   │   ├── cine_sax_ks.mat
│   │   ├── t1map_calib.mat
│   │   ├── t1map_info.csv
│   │   ├── t1map_ks.mat
│   │   ├── t2map_calib.mat
│   │   ├── t2map_info.csv
│   │   └── t2map_ks.mat
│   ├── P070
│   │   ├── t1map_calib.mat
│   │   ├── t1map_info.csv
│   │   ├── t1map_ks.mat
│   │   ├── t2map_calib.mat
│   │   ├── t2map_info.csv
│   │   └── t2map_ks.mat
│   ├── P071
│   │   ├── cine_lax_calib.mat
│   │   ├── cine_lax_info.csv
│   │   ├── cine_lax_ks.mat
│   │   ├── cine_sax_calib.mat
│   │   ├── cine_sax_info.csv
│   │   ├── cine_sax_ks.mat
│   │   ├── t1map_calib.mat
│   │   ├── t1map_info.csv
│   │   ├── t1map_ks.mat
```

```
│       │   ├── t2map_calib.mat
│       │   ├── t2map_info.csv
│       │   └── t2map_ks.mat
│       ├── P072
│       │   ├── cine_lax_calib.mat
│       │   ├── cine_lax_info.csv
│       │   ├── cine_lax_ks.mat
│       │   ├── cine_sax_calib.mat
│       │   ├── cine_sax_info.csv
│       │   ├── cine_sax_ks.mat
│       │   ├── t1map_calib.mat
│       │   ├── t1map_info.csv
│       │   ├── t1map_ks.mat
│       │   ├── t2map_calib.mat
│       │   ├── t2map_info.csv
│       │   └── t2map_ks.mat
│       ├── P073
│       │   ├── cine_lax_calib.mat
│       │   ├── cine_lax_info.csv
│       │   ├── cine_lax_ks.mat
│       │   ├── cine_sax_calib.mat
│       │   ├── cine_sax_info.csv
│       │   ├── cine_sax_ks.mat
│       │   ├── t1map_calib.mat
│       │   ├── t1map_info.csv
│       │   ├── t1map_ks.mat
│       │   ├── t2map_calib.mat
│       │   ├── t2map_info.csv
│       │   └── t2map_ks.mat
│       ├── P074
│       │   ├── cine_lax_calib.mat
│       │   ├── cine_lax_info.csv
│       │   ├── cine_lax_ks.mat
│       │   ├── t1map_calib.mat
│       │   ├── t1map_info.csv
│       │   ├── t1map_ks.mat
│       │   ├── t2map_calib.mat
│       │   ├── t2map_info.csv
│       │   └── t2map_ks.mat
│       ├── P075
│       │   ├── cine_lax_calib.mat
│       │   ├── cine_lax_info.csv
│       │   ├── cine_lax_ks.mat
│       │   ├── cine_sax_calib.mat
│       │   ├── cine_sax_info.csv
│       │   ├── cine_sax_ks.mat
│       │   ├── t1map_calib.mat
│       │   ├── t1map_info.csv
│       │   ├── t1map_ks.mat
│       │   ├── t2map_calib.mat
│       │   ├── t2map_info.csv
│       │   └── t2map_ks.mat
│       ├── P076
│       │   ├── cine_lax_calib.mat
```

```
│       │       ├── cine_lax_info.csv
│       │       ├── cine_lax_ks.mat
│       │       ├── cine_sax_calib.mat
│       │       ├── cine_sax_info.csv
│       │       ├── cine_sax_ks.mat
│       │       ├── t1map_calib.mat
│       │       ├── t1map_info.csv
│       │       ├── t1map_ks.mat
│       │       ├── t2map_calib.mat
│       │       ├── t2map_info.csv
│       │       └── t2map_ks.mat
│       ├── P077
│       │       ├── cine_lax_calib.mat
│       │       ├── cine_lax_info.csv
│       │       ├── cine_lax_ks.mat
│       │       ├── cine_sax_calib.mat
│       │       ├── cine_sax_info.csv
│       │       ├── cine_sax_ks.mat
│       │       ├── t1map_calib.mat
│       │       ├── t1map_info.csv
│       │       ├── t1map_ks.mat
│       │       ├── t2map_calib.mat
│       │       ├── t2map_info.csv
│       │       └── t2map_ks.mat
│       ├── P078
│       │       ├── cine_lax_calib.mat
│       │       ├── cine_lax_info.csv
│       │       ├── cine_lax_ks.mat
│       │       ├── cine_sax_calib.mat
│       │       ├── cine_sax_info.csv
│       │       ├── cine_sax_ks.mat
│       │       ├── t1map_calib.mat
│       │       ├── t1map_info.csv
│       │       ├── t1map_ks.mat
│       │       ├── t2map_calib.mat
│       │       ├── t2map_info.csv
│       │       └── t2map_ks.mat
│       ├── P079
│       │       ├── cine_lax_calib.mat
│       │       ├── cine_lax_info.csv
│       │       ├── cine_lax_ks.mat
│       │       ├── cine_sax_calib.mat
│       │       ├── cine_sax_info.csv
│       │       ├── cine_sax_ks.mat
│       │       ├── t1map_calib.mat
│       │       ├── t1map_info.csv
│       │       ├── t1map_ks.mat
│       │       ├── t2map_calib.mat
│       │       ├── t2map_info.csv
│       │       └── t2map_ks.mat
│       ├── P080
│       │       ├── cine_lax_calib.mat
│       │       ├── cine_lax_info.csv
│       │       ├── cine_lax_ks.mat
```

```
│       │    ├── t1map_calib.mat
│       │    ├── t1map_info.csv
│       │    ├── t1map_ks.mat
│       │    ├── t2map_calib.mat
│       │    ├── t2map_info.csv
│       │    └── t2map_ks.mat
│       ├── P081
│       │    ├── cine_lax_calib.mat
│       │    ├── cine_lax_info.csv
│       │    ├── cine_lax_ks.mat
│       │    ├── cine_sax_calib.mat
│       │    ├── cine_sax_info.csv
│       │    ├── cine_sax_ks.mat
│       │    ├── t1map_calib.mat
│       │    ├── t1map_info.csv
│       │    ├── t1map_ks.mat
│       │    ├── t2map_calib.mat
│       │    ├── t2map_info.csv
│       │    └── t2map_ks.mat
│       ├── P082
│       │    ├── cine_lax_calib.mat
│       │    ├── cine_lax_info.csv
│       │    ├── cine_lax_ks.mat
│       │    ├── cine_sax_calib.mat
│       │    ├── cine_sax_info.csv
│       │    ├── cine_sax_ks.mat
│       │    ├── t1map_calib.mat
│       │    ├── t1map_info.csv
│       │    ├── t1map_ks.mat
│       │    ├── t2map_calib.mat
│       │    ├── t2map_info.csv
│       │    └── t2map_ks.mat
│       ├── P083
│       │    ├── t1map_calib.mat
│       │    ├── t1map_info.csv
│       │    ├── t1map_ks.mat
│       │    ├── t2map_calib.mat
│       │    ├── t2map_info.csv
│       │    └── t2map_ks.mat
│       ├── P084
│       │    ├── cine_lax_calib.mat
│       │    ├── cine_lax_info.csv
│       │    ├── cine_lax_ks.mat
│       │    ├── cine_sax_calib.mat
│       │    ├── cine_sax_info.csv
│       │    ├── cine_sax_ks.mat
│       │    ├── t1map_calib.mat
│       │    ├── t1map_info.csv
│       │    ├── t1map_ks.mat
│       │    ├── t2map_calib.mat
│       │    ├── t2map_info.csv
│       │    └── t2map_ks.mat
│       ├── P085
│       │    ├── t1map_calib.mat
```

```
│       │   ├── t1map_info.csv
│       │   ├── t1map_ks.mat
│       │   ├── t2map_calib.mat
│       │   ├── t2map_info.csv
│       │   └── t2map_ks.mat
│       ├── P086
│       │   ├── cine_lax_calib.mat
│       │   ├── cine_lax_info.csv
│       │   ├── cine_lax_ks.mat
│       │   ├── cine_sax_calib.mat
│       │   ├── cine_sax_info.csv
│       │   ├── cine_sax_ks.mat
│       │   ├── t1map_calib.mat
│       │   ├── t1map_info.csv
│       │   ├── t1map_ks.mat
│       │   ├── t2map_calib.mat
│       │   ├── t2map_info.csv
│       │   └── t2map_ks.mat
│       ├── P087
│       │   ├── cine_lax_calib.mat
│       │   ├── cine_lax_info.csv
│       │   ├── cine_lax_ks.mat
│       │   ├── cine_sax_calib.mat
│       │   ├── cine_sax_info.csv
│       │   ├── cine_sax_ks.mat
│       │   ├── t1map_calib.mat
│       │   ├── t1map_info.csv
│       │   ├── t1map_ks.mat
│       │   ├── t2map_calib.mat
│       │   ├── t2map_info.csv
│       │   └── t2map_ks.mat
│       ├── P088
│       │   ├── cine_lax_calib.mat
│       │   ├── cine_lax_info.csv
│       │   ├── cine_lax_ks.mat
│       │   ├── cine_sax_calib.mat
│       │   ├── cine_sax_info.csv
│       │   ├── cine_sax_ks.mat
│       │   ├── t1map_calib.mat
│       │   ├── t1map_info.csv
│       │   ├── t1map_ks.mat
│       │   ├── t2map_calib.mat
│       │   ├── t2map_info.csv
│       │   └── t2map_ks.mat
│       ├── P089
│       │   ├── cine_lax_calib.mat
│       │   ├── cine_lax_info.csv
│       │   ├── cine_lax_ks.mat
│       │   ├── cine_sax_calib.mat
│       │   ├── cine_sax_info.csv
│       │   ├── cine_sax_ks.mat
│       │   ├── t1map_calib.mat
│       │   ├── t1map_info.csv
│       │   ├── t1map_ks.mat
```

```
│       │       ├── t2map_calib.mat
│       │       ├── t2map_info.csv
│       │       └── t2map_ks.mat
│       ├── P090
│       │       ├── cine_lax_calib.mat
│       │       ├── cine_lax_info.csv
│       │       ├── cine_lax_ks.mat
│       │       ├── cine_sax_calib.mat
│       │       ├── cine_sax_info.csv
│       │       ├── cine_sax_ks.mat
│       │       ├── t1map_calib.mat
│       │       ├── t1map_info.csv
│       │       ├── t1map_ks.mat
│       │       ├── t2map_calib.mat
│       │       ├── t2map_info.csv
│       │       └── t2map_ks.mat
│       ├── P091
│       │       ├── cine_lax_calib.mat
│       │       ├── cine_lax_info.csv
│       │       ├── cine_lax_ks.mat
│       │       ├── cine_sax_calib.mat
│       │       ├── cine_sax_info.csv
│       │       ├── cine_sax_ks.mat
│       │       ├── t1map_calib.mat
│       │       ├── t1map_info.csv
│       │       ├── t1map_ks.mat
│       │       ├── t2map_calib.mat
│       │       ├── t2map_info.csv
│       │       └── t2map_ks.mat
│       ├── P092
│       │       ├── cine_lax_calib.mat
│       │       ├── cine_lax_info.csv
│       │       ├── cine_lax_ks.mat
│       │       ├── cine_sax_calib.mat
│       │       ├── cine_sax_info.csv
│       │       ├── cine_sax_ks.mat
│       │       ├── t1map_calib.mat
│       │       ├── t1map_info.csv
│       │       ├── t1map_ks.mat
│       │       ├── t2map_calib.mat
│       │       ├── t2map_info.csv
│       │       └── t2map_ks.mat
│       ├── P093
│       │       ├── cine_lax_calib.mat
│       │       ├── cine_lax_info.csv
│       │       ├── cine_lax_ks.mat
│       │       ├── cine_sax_calib.mat
│       │       ├── cine_sax_info.csv
│       │       ├── cine_sax_ks.mat
│       │       ├── t1map_calib.mat
│       │       ├── t1map_info.csv
│       │       ├── t1map_ks.mat
│       │       ├── t2map_calib.mat
│       │       ├── t2map_info.csv
```

```
│       │     └── t2map_ks.mat
│       ├── P094
│       │     ├── cine_lax_calib.mat
│       │     ├── cine_lax_info.csv
│       │     ├── cine_lax_ks.mat
│       │     ├── cine_sax_calib.mat
│       │     ├── cine_sax_info.csv
│       │     ├── cine_sax_ks.mat
│       │     ├── t1map_calib.mat
│       │     ├── t1map_info.csv
│       │     ├── t1map_ks.mat
│       │     ├── t2map_calib.mat
│       │     ├── t2map_info.csv
│       │     └── t2map_ks.mat
│       ├── P095
│       │     ├── cine_lax_calib.mat
│       │     ├── cine_lax_info.csv
│       │     ├── cine_lax_ks.mat
│       │     ├── cine_sax_calib.mat
│       │     ├── cine_sax_info.csv
│       │     ├── cine_sax_ks.mat
│       │     ├── t1map_calib.mat
│       │     ├── t1map_info.csv
│       │     ├── t1map_ks.mat
│       │     ├── t2map_calib.mat
│       │     ├── t2map_info.csv
│       │     └── t2map_ks.mat
│       ├── P096
│       │     ├── cine_lax_calib.mat
│       │     ├── cine_lax_info.csv
│       │     ├── cine_lax_ks.mat
│       │     ├── cine_sax_calib.mat
│       │     ├── cine_sax_info.csv
│       │     ├── cine_sax_ks.mat
│       │     ├── t1map_calib.mat
│       │     ├── t1map_info.csv
│       │     ├── t1map_ks.mat
│       │     ├── t2map_calib.mat
│       │     ├── t2map_info.csv
│       │     └── t2map_ks.mat
│       ├── P097
│       │     ├── cine_lax_calib.mat
│       │     ├── cine_lax_info.csv
│       │     ├── cine_lax_ks.mat
│       │     ├── cine_sax_calib.mat
│       │     ├── cine_sax_info.csv
│       │     ├── cine_sax_ks.mat
│       │     ├── t1map_calib.mat
│       │     ├── t1map_info.csv
│       │     ├── t1map_ks.mat
│       │     ├── t2map_calib.mat
│       │     ├── t2map_info.csv
│       │     └── t2map_ks.mat
│       ├── P098
```

```
│       │   ├── cine_lax_calib.mat
│       │   ├── cine_lax_info.csv
│       │   ├── cine_lax_ks.mat
│       │   ├── cine_sax_calib.mat
│       │   ├── cine_sax_info.csv
│       │   ├── cine_sax_ks.mat
│       │   ├── t1map_calib.mat
│       │   ├── t1map_info.csv
│       │   ├── t1map_ks.mat
│       │   ├── t2map_calib.mat
│       │   ├── t2map_info.csv
│       │   └── t2map_ks.mat
│       ├── P099
│       │   ├── cine_lax_calib.mat
│       │   ├── cine_lax_info.csv
│       │   ├── cine_lax_ks.mat
│       │   ├── cine_sax_calib.mat
│       │   ├── cine_sax_info.csv
│       │   ├── cine_sax_ks.mat
│       │   ├── t1map_calib.mat
│       │   ├── t1map_info.csv
│       │   ├── t1map_ks.mat
│       │   ├── t2map_calib.mat
│       │   ├── t2map_info.csv
│       │   └── t2map_ks.mat
│       ├── P100
│       │   ├── cine_lax_calib.mat
│       │   ├── cine_lax_info.csv
│       │   ├── cine_lax_ks.mat
│       │   ├── cine_sax_calib.mat
│       │   ├── cine_sax_info.csv
│       │   ├── cine_sax_ks.mat
│       │   ├── t1map_calib.mat
│       │   ├── t1map_info.csv
│       │   ├── t1map_ks.mat
│       │   ├── t2map_calib.mat
│       │   ├── t2map_info.csv
│       │   └── t2map_ks.mat
│       ├── P101
│       │   ├── cine_lax_calib.mat
│       │   ├── cine_lax_info.csv
│       │   ├── cine_lax_ks.mat
│       │   ├── cine_sax_calib.mat
│       │   ├── cine_sax_info.csv
│       │   ├── cine_sax_ks.mat
│       │   ├── t1map_calib.mat
│       │   ├── t1map_info.csv
│       │   ├── t1map_ks.mat
│       │   ├── t2map_calib.mat
│       │   ├── t2map_info.csv
│       │   └── t2map_ks.mat
│       ├── P102
│       │   ├── cine_lax_calib.mat
│       │   ├── cine_lax_info.csv
```

```
│       │    ├── cine_lax_ks.mat
│       │    ├── cine_sax_calib.mat
│       │    ├── cine_sax_info.csv
│       │    ├── cine_sax_ks.mat
│       │    ├── t1map_calib.mat
│       │    ├── t1map_info.csv
│       │    ├── t1map_ks.mat
│       │    ├── t2map_calib.mat
│       │    ├── t2map_info.csv
│       │    └── t2map_ks.mat
│       ├── P103
│       │    ├── cine_lax_calib.mat
│       │    ├── cine_lax_info.csv
│       │    ├── cine_lax_ks.mat
│       │    ├── cine_sax_calib.mat
│       │    ├── cine_sax_info.csv
│       │    ├── cine_sax_ks.mat
│       │    ├── t1map_calib.mat
│       │    ├── t1map_info.csv
│       │    ├── t1map_ks.mat
│       │    ├── t2map_calib.mat
│       │    ├── t2map_info.csv
│       │    └── t2map_ks.mat
│       ├── P104
│       │    ├── cine_lax_calib.mat
│       │    ├── cine_lax_info.csv
│       │    ├── cine_lax_ks.mat
│       │    ├── cine_sax_calib.mat
│       │    ├── cine_sax_info.csv
│       │    ├── cine_sax_ks.mat
│       │    ├── t1map_calib.mat
│       │    ├── t1map_info.csv
│       │    ├── t1map_ks.mat
│       │    ├── t2map_calib.mat
│       │    ├── t2map_info.csv
│       │    └── t2map_ks.mat
│       ├── P105
│       │    ├── t1map_calib.mat
│       │    ├── t1map_info.csv
│       │    ├── t1map_ks.mat
│       │    ├── t2map_calib.mat
│       │    ├── t2map_info.csv
│       │    └── t2map_ks.mat
│       ├── P106
│       │    ├── t1map_calib.mat
│       │    ├── t1map_info.csv
│       │    ├── t1map_ks.mat
│       │    ├── t2map_calib.mat
│       │    ├── t2map_info.csv
│       │    └── t2map_ks.mat
│       ├── P107
│       │    ├── cine_sax_calib.mat
│       │    ├── cine_sax_info.csv
│       │    ├── cine_sax_ks.mat
```

```
│   │   ├── t1map_calib.mat
│   │   ├── t1map_info.csv
│   │   ├── t1map_ks.mat
│   │   ├── t2map_calib.mat
│   │   ├── t2map_info.csv
│   │   └── t2map_ks.mat
│   ├── P108
│   │   ├── cine_sax_calib.mat
│   │   ├── cine_sax_info.csv
│   │   ├── cine_sax_ks.mat
│   │   ├── t1map_calib.mat
│   │   ├── t1map_info.csv
│   │   ├── t1map_ks.mat
│   │   ├── t2map_calib.mat
│   │   ├── t2map_info.csv
│   │   └── t2map_ks.mat
│   ├── P109
│   │   ├── cine_sax_calib.mat
│   │   ├── cine_sax_info.csv
│   │   ├── cine_sax_ks.mat
│   │   ├── t1map_calib.mat
│   │   ├── t1map_info.csv
│   │   ├── t1map_ks.mat
│   │   ├── t2map_calib.mat
│   │   ├── t2map_info.csv
│   │   └── t2map_ks.mat
│   ├── P110
│   │   ├── cine_sax_calib.mat
│   │   ├── cine_sax_info.csv
│   │   ├── cine_sax_ks.mat
│   │   ├── t1map_calib.mat
│   │   ├── t1map_info.csv
│   │   ├── t1map_ks.mat
│   │   ├── t2map_calib.mat
│   │   ├── t2map_info.csv
│   │   └── t2map_ks.mat
│   ├── P111
│   │   ├── t1map_calib.mat
│   │   ├── t1map_info.csv
│   │   ├── t1map_ks.mat
│   │   ├── t2map_calib.mat
│   │   ├── t2map_info.csv
│   │   └── t2map_ks.mat
│   ├── P112
│   │   ├── t1map_calib.mat
│   │   ├── t1map_info.csv
│   │   ├── t1map_ks.mat
│   │   ├── t2map_calib.mat
│   │   ├── t2map_info.csv
│   │   └── t2map_ks.mat
│   ├── P113
│   │   ├── cine_lax_calib.mat
│   │   ├── cine_lax_info.csv
│   │   ├── cine_lax_ks.mat
```

```
│       │       ├── t1map_calib.mat
│       │       ├── t1map_info.csv
│       │       ├── t1map_ks.mat
│       │       ├── t2map_calib.mat
│       │       ├── t2map_info.csv
│       │       └── t2map_ks.mat
│       ├── P114
│       │       ├── t1map_calib.mat
│       │       ├── t1map_info.csv
│       │       ├── t1map_ks.mat
│       │       ├── t2map_calib.mat
│       │       ├── t2map_info.csv
│       │       └── t2map_ks.mat
│       ├── P115
│       │       ├── cine_sax_calib.mat
│       │       ├── cine_sax_info.csv
│       │       ├── cine_sax_ks.mat
│       │       ├── t1map_calib.mat
│       │       ├── t1map_info.csv
│       │       ├── t1map_ks.mat
│       │       ├── t2map_calib.mat
│       │       ├── t2map_info.csv
│       │       └── t2map_ks.mat
│       ├── P116
│       │       ├── cine_sax_calib.mat
│       │       ├── cine_sax_info.csv
│       │       ├── cine_sax_ks.mat
│       │       ├── t1map_calib.mat
│       │       ├── t1map_info.csv
│       │       ├── t1map_ks.mat
│       │       ├── t2map_calib.mat
│       │       ├── t2map_info.csv
│       │       └── t2map_ks.mat
│       ├── P117
│       │       ├── cine_lax_calib.mat
│       │       ├── cine_lax_info.csv
│       │       ├── cine_lax_ks.mat
│       │       ├── t1map_calib.mat
│       │       ├── t1map_info.csv
│       │       ├── t1map_ks.mat
│       │       ├── t2map_calib.mat
│       │       ├── t2map_info.csv
│       │       └── t2map_ks.mat
│       ├── P118
│       │       ├── cine_lax_calib.mat
│       │       ├── cine_lax_info.csv
│       │       ├── cine_lax_ks.mat
│       │       ├── cine_sax_calib.mat
│       │       ├── cine_sax_info.csv
│       │       ├── cine_sax_ks.mat
│       │       ├── t1map_calib.mat
│       │       ├── t1map_info.csv
│       │       ├── t1map_ks.mat
│       │       ├── t2map_calib.mat
```

```
│       │       ├── t2map_info.csv
│       │       └── t2map_ks.mat
│       ├── P119
│       │       ├── cine_sax_calib.mat
│       │       ├── cine_sax_info.csv
│       │       ├── cine_sax_ks.mat
│       │       ├── t1map_calib.mat
│       │       ├── t1map_info.csv
│       │       ├── t1map_ks.mat
│       │       ├── t2map_calib.mat
│       │       ├── t2map_info.csv
│       │       └── t2map_ks.mat
│       └── P120
│               ├── t1map_calib.mat
│               ├── t1map_info.csv
│               ├── t1map_ks.mat
│               ├── t2map_calib.mat
│               ├── t2map_info.csv
│               └── t2map_ks.mat
├── TrainingSet
│       ├── P001
│       │       ├── cine_lax_calib.mat
│       │       ├── cine_lax_info.csv
│       │       ├── cine_lax_ks.mat
│       │       ├── cine_sax_calib.mat
│       │       ├── cine_sax_info.csv
│       │       ├── cine_sax_ks.mat
│       │       ├── t1map_calib.mat
│       │       ├── t1map_info.csv
│       │       ├── t1map_ks.mat
│       │       ├── t2map_calib.mat
│       │       ├── t2map_info.csv
│       │       └── t2map_ks.mat
│       ├── P002
│       │       ├── cine_lax_calib.mat
│       │       ├── cine_lax_info.csv
│       │       ├── cine_lax_ks.mat
│       │       ├── cine_sax_calib.mat
│       │       ├── cine_sax_info.csv
│       │       ├── cine_sax_ks.mat
│       │       ├── t1map_calib.mat
│       │       ├── t1map_info.csv
│       │       ├── t1map_ks.mat
│       │       ├── t2map_calib.mat
│       │       ├── t2map_info.csv
│       │       └── t2map_ks.mat
│       ├── P003
│       │       ├── cine_lax_calib.mat
│       │       ├── cine_lax_info.csv
│       │       ├── cine_lax_ks.mat
│       │       ├── cine_sax_calib.mat
│       │       ├── cine_sax_info.csv
│       │       ├── cine_sax_ks.mat
│       │       ├── t1map_calib.mat
```

```
│       │       ├── t1map_info.csv
│       │       ├── t1map_ks.mat
│       │       ├── t2map_calib.mat
│       │       ├── t2map_info.csv
│       │       └── t2map_ks.mat
│       ├── P004
│       │       ├── cine_lax_calib.mat
│       │       ├── cine_lax_info.csv
│       │       ├── cine_lax_ks.mat
│       │       ├── cine_sax_calib.mat
│       │       ├── cine_sax_info.csv
│       │       ├── cine_sax_ks.mat
│       │       ├── t1map_calib.mat
│       │       ├── t1map_info.csv
│       │       ├── t1map_ks.mat
│       │       ├── t2map_calib.mat
│       │       ├── t2map_info.csv
│       │       └── t2map_ks.mat
│       ├── P005
│       │       ├── cine_lax_calib.mat
│       │       ├── cine_lax_info.csv
│       │       ├── cine_lax_ks.mat
│       │       ├── cine_sax_calib.mat
│       │       ├── cine_sax_info.csv
│       │       ├── cine_sax_ks.mat
│       │       ├── t1map_calib.mat
│       │       ├── t1map_info.csv
│       │       ├── t1map_ks.mat
│       │       ├── t2map_calib.mat
│       │       ├── t2map_info.csv
│       │       └── t2map_ks.mat
│       ├── P006
│       │       ├── cine_lax_calib.mat
│       │       ├── cine_lax_info.csv
│       │       ├── cine_lax_ks.mat
│       │       ├── cine_sax_calib.mat
│       │       ├── cine_sax_info.csv
│       │       ├── cine_sax_ks.mat
│       │       ├── t1map_calib.mat
│       │       ├── t1map_info.csv
│       │       ├── t1map_ks.mat
│       │       ├── t2map_calib.mat
│       │       ├── t2map_info.csv
│       │       └── t2map_ks.mat
│       ├── P007
│       │       ├── cine_lax_calib.mat
│       │       ├── cine_lax_info.csv
│       │       ├── cine_lax_ks.mat
│       │       ├── cine_sax_calib.mat
│       │       ├── cine_sax_info.csv
│       │       ├── cine_sax_ks.mat
│       │       ├── t1map_calib.mat
│       │       ├── t1map_info.csv
│       │       ├── t1map_ks.mat
```

```
│       │       ├── t2map_calib.mat
│       │       ├── t2map_info.csv
│       │       └── t2map_ks.mat
│       ├── P008
│       │       ├── cine_lax_calib.mat
│       │       ├── cine_lax_info.csv
│       │       ├── cine_lax_ks.mat
│       │       ├── cine_sax_calib.mat
│       │       ├── cine_sax_info.csv
│       │       ├── cine_sax_ks.mat
│       │       ├── t1map_calib.mat
│       │       ├── t1map_info.csv
│       │       ├── t1map_ks.mat
│       │       ├── t2map_calib.mat
│       │       ├── t2map_info.csv
│       │       └── t2map_ks.mat
│       ├── P009
│       │       ├── cine_lax_calib.mat
│       │       ├── cine_lax_info.csv
│       │       ├── cine_lax_ks.mat
│       │       ├── cine_sax_calib.mat
│       │       ├── cine_sax_info.csv
│       │       ├── cine_sax_ks.mat
│       │       ├── t1map_calib.mat
│       │       ├── t1map_info.csv
│       │       ├── t1map_ks.mat
│       │       ├── t2map_calib.mat
│       │       ├── t2map_info.csv
│       │       └── t2map_ks.mat
│       ├── P010
│       │       ├── cine_lax_calib.mat
│       │       ├── cine_lax_info.csv
│       │       ├── cine_lax_ks.mat
│       │       ├── cine_sax_calib.mat
│       │       ├── cine_sax_info.csv
│       │       ├── cine_sax_ks.mat
│       │       ├── t1map_calib.mat
│       │       ├── t1map_info.csv
│       │       ├── t1map_ks.mat
│       │       ├── t2map_calib.mat
│       │       ├── t2map_info.csv
│       │       └── t2map_ks.mat
│       ├── P011
│       │       ├── cine_lax_calib.mat
│       │       ├── cine_lax_info.csv
│       │       ├── cine_lax_ks.mat
│       │       ├── cine_sax_calib.mat
│       │       ├── cine_sax_info.csv
│       │       ├── cine_sax_ks.mat
│       │       ├── t1map_calib.mat
│       │       ├── t1map_info.csv
│       │       ├── t1map_ks.mat
│       │       ├── t2map_calib.mat
│       │       ├── t2map_info.csv
```

```
│       │   └── t2map_ks.mat
│       ├── P012
│       │   ├── cine_lax_calib.mat
│       │   ├── cine_lax_info.csv
│       │   ├── cine_lax_ks.mat
│       │   ├── cine_sax_calib.mat
│       │   ├── cine_sax_info.csv
│       │   ├── cine_sax_ks.mat
│       │   ├── t1map_calib.mat
│       │   ├── t1map_info.csv
│       │   ├── t1map_ks.mat
│       │   ├── t2map_calib.mat
│       │   ├── t2map_info.csv
│       │   └── t2map_ks.mat
│       ├── P013
│       │   ├── cine_lax_calib.mat
│       │   ├── cine_lax_info.csv
│       │   ├── cine_lax_ks.mat
│       │   ├── cine_sax_calib.mat
│       │   ├── cine_sax_info.csv
│       │   ├── cine_sax_ks.mat
│       │   ├── t1map_calib.mat
│       │   ├── t1map_info.csv
│       │   ├── t1map_ks.mat
│       │   ├── t2map_calib.mat
│       │   ├── t2map_info.csv
│       │   └── t2map_ks.mat
│       ├── P014
│       │   ├── cine_lax_calib.mat
│       │   ├── cine_lax_info.csv
│       │   ├── cine_lax_ks.mat
│       │   ├── cine_sax_calib.mat
│       │   ├── cine_sax_info.csv
│       │   ├── cine_sax_ks.mat
│       │   ├── t1map_calib.mat
│       │   ├── t1map_info.csv
│       │   ├── t1map_ks.mat
│       │   ├── t2map_calib.mat
│       │   ├── t2map_info.csv
│       │   └── t2map_ks.mat
│       ├── P015
│       │   ├── cine_lax_calib.mat
│       │   ├── cine_lax_info.csv
│       │   ├── cine_lax_ks.mat
│       │   ├── cine_sax_calib.mat
│       │   ├── cine_sax_info.csv
│       │   ├── cine_sax_ks.mat
│       │   ├── t1map_calib.mat
│       │   ├── t1map_info.csv
│       │   ├── t1map_ks.mat
│       │   ├── t2map_calib.mat
│       │   ├── t2map_info.csv
│       │   └── t2map_ks.mat
│       ├── P016
```

```
│       │       ├── cine_lax_calib.mat
│       │       ├── cine_lax_info.csv
│       │       ├── cine_lax_ks.mat
│       │       ├── cine_sax_calib.mat
│       │       ├── cine_sax_info.csv
│       │       ├── cine_sax_ks.mat
│       │       ├── t1map_calib.mat
│       │       ├── t1map_info.csv
│       │       ├── t1map_ks.mat
│       │       ├── t2map_calib.mat
│       │       ├── t2map_info.csv
│       │       └── t2map_ks.mat
│       ├── P017
│       │       ├── cine_lax_calib.mat
│       │       ├── cine_lax_info.csv
│       │       ├── cine_lax_ks.mat
│       │       ├── cine_sax_calib.mat
│       │       ├── cine_sax_info.csv
│       │       ├── cine_sax_ks.mat
│       │       ├── t1map_calib.mat
│       │       ├── t1map_info.csv
│       │       ├── t1map_ks.mat
│       │       ├── t2map_calib.mat
│       │       ├── t2map_info.csv
│       │       └── t2map_ks.mat
│       ├── P018
│       │       ├── cine_lax_calib.mat
│       │       ├── cine_lax_info.csv
│       │       ├── cine_lax_ks.mat
│       │       ├── cine_sax_calib.mat
│       │       ├── cine_sax_info.csv
│       │       ├── cine_sax_ks.mat
│       │       ├── t1map_calib.mat
│       │       ├── t1map_info.csv
│       │       ├── t1map_ks.mat
│       │       ├── t2map_calib.mat
│       │       ├── t2map_info.csv
│       │       └── t2map_ks.mat
│       ├── P019
│       │       ├── cine_lax_calib.mat
│       │       ├── cine_lax_info.csv
│       │       ├── cine_lax_ks.mat
│       │       ├── cine_sax_calib.mat
│       │       ├── cine_sax_info.csv
│       │       ├── cine_sax_ks.mat
│       │       ├── t1map_calib.mat
│       │       ├── t1map_info.csv
│       │       ├── t1map_ks.mat
│       │       ├── t2map_calib.mat
│       │       ├── t2map_info.csv
│       │       └── t2map_ks.mat
│       ├── P020
│       │       ├── cine_lax_calib.mat
│       │       ├── cine_lax_info.csv
```

```
│       │       ├── cine_lax_ks.mat
│       │       ├── cine_sax_calib.mat
│       │       ├── cine_sax_info.csv
│       │       ├── cine_sax_ks.mat
│       │       ├── t1map_calib.mat
│       │       ├── t1map_info.csv
│       │       ├── t1map_ks.mat
│       │       ├── t2map_calib.mat
│       │       ├── t2map_info.csv
│       │       └── t2map_ks.mat
│       ├── P021
│       │       ├── cine_lax_calib.mat
│       │       ├── cine_lax_info.csv
│       │       ├── cine_lax_ks.mat
│       │       ├── cine_sax_calib.mat
│       │       ├── cine_sax_info.csv
│       │       ├── cine_sax_ks.mat
│       │       ├── t1map_calib.mat
│       │       ├── t1map_info.csv
│       │       ├── t1map_ks.mat
│       │       ├── t2map_calib.mat
│       │       ├── t2map_info.csv
│       │       └── t2map_ks.mat
│       ├── P022
│       │       ├── cine_lax_calib.mat
│       │       ├── cine_lax_info.csv
│       │       ├── cine_lax_ks.mat
│       │       ├── cine_sax_calib.mat
│       │       ├── cine_sax_info.csv
│       │       ├── cine_sax_ks.mat
│       │       ├── t1map_calib.mat
│       │       ├── t1map_info.csv
│       │       ├── t1map_ks.mat
│       │       ├── t2map_calib.mat
│       │       ├── t2map_info.csv
│       │       └── t2map_ks.mat
│       ├── P023
│       │       ├── cine_lax_calib.mat
│       │       ├── cine_lax_info.csv
│       │       ├── cine_lax_ks.mat
│       │       ├── cine_sax_calib.mat
│       │       ├── cine_sax_info.csv
│       │       ├── cine_sax_ks.mat
│       │       ├── t1map_calib.mat
│       │       ├── t1map_info.csv
│       │       ├── t1map_ks.mat
│       │       ├── t2map_calib.mat
│       │       ├── t2map_info.csv
│       │       └── t2map_ks.mat
│       ├── P024
│       │       ├── cine_lax_calib.mat
│       │       ├── cine_lax_info.csv
│       │       ├── cine_lax_ks.mat
│       │       ├── cine_sax_calib.mat
```

```
│       │       ├── cine_sax_info.csv
│       │       ├── cine_sax_ks.mat
│       │       ├── t1map_calib.mat
│       │       ├── t1map_info.csv
│       │       ├── t1map_ks.mat
│       │       ├── t2map_calib.mat
│       │       ├── t2map_info.csv
│       │       └── t2map_ks.mat
│       ├── P025
│       │       ├── cine_lax_calib.mat
│       │       ├── cine_lax_info.csv
│       │       ├── cine_lax_ks.mat
│       │       ├── cine_sax_calib.mat
│       │       ├── cine_sax_info.csv
│       │       ├── cine_sax_ks.mat
│       │       ├── t1map_calib.mat
│       │       ├── t1map_info.csv
│       │       ├── t1map_ks.mat
│       │       ├── t2map_calib.mat
│       │       ├── t2map_info.csv
│       │       └── t2map_ks.mat
│       ├── P026
│       │       ├── cine_lax_calib.mat
│       │       ├── cine_lax_info.csv
│       │       ├── cine_lax_ks.mat
│       │       ├── cine_sax_calib.mat
│       │       ├── cine_sax_info.csv
│       │       ├── cine_sax_ks.mat
│       │       ├── t1map_calib.mat
│       │       ├── t1map_info.csv
│       │       ├── t1map_ks.mat
│       │       ├── t2map_calib.mat
│       │       ├── t2map_info.csv
│       │       └── t2map_ks.mat
│       ├── P027
│       │       ├── cine_lax_calib.mat
│       │       ├── cine_lax_info.csv
│       │       ├── cine_lax_ks.mat
│       │       ├── cine_sax_calib.mat
│       │       ├── cine_sax_info.csv
│       │       ├── cine_sax_ks.mat
│       │       ├── t1map_calib.mat
│       │       ├── t1map_info.csv
│       │       ├── t1map_ks.mat
│       │       ├── t2map_calib.mat
│       │       ├── t2map_info.csv
│       │       └── t2map_ks.mat
│       ├── P028
│       │       ├── cine_lax_calib.mat
│       │       ├── cine_lax_info.csv
│       │       ├── cine_lax_ks.mat
│       │       ├── cine_sax_calib.mat
│       │       ├── cine_sax_info.csv
│       │       ├── cine_sax_ks.mat
```

```
│       │       ├── t1map_calib.mat
│       │       ├── t1map_info.csv
│       │       ├── t1map_ks.mat
│       │       ├── t2map_calib.mat
│       │       ├── t2map_info.csv
│       │       └── t2map_ks.mat
│       ├── P029
│       │       ├── cine_lax_calib.mat
│       │       ├── cine_lax_info.csv
│       │       ├── cine_lax_ks.mat
│       │       ├── cine_sax_calib.mat
│       │       ├── cine_sax_info.csv
│       │       ├── cine_sax_ks.mat
│       │       ├── t1map_calib.mat
│       │       ├── t1map_info.csv
│       │       ├── t1map_ks.mat
│       │       ├── t2map_calib.mat
│       │       ├── t2map_info.csv
│       │       └── t2map_ks.mat
│       ├── P030
│       │       ├── cine_lax_calib.mat
│       │       ├── cine_lax_info.csv
│       │       ├── cine_lax_ks.mat
│       │       ├── cine_sax_calib.mat
│       │       ├── cine_sax_info.csv
│       │       ├── cine_sax_ks.mat
│       │       ├── t1map_calib.mat
│       │       ├── t1map_info.csv
│       │       ├── t1map_ks.mat
│       │       ├── t2map_calib.mat
│       │       ├── t2map_info.csv
│       │       └── t2map_ks.mat
│       ├── P031
│       │       ├── cine_lax_calib.mat
│       │       ├── cine_lax_info.csv
│       │       ├── cine_lax_ks.mat
│       │       ├── cine_sax_calib.mat
│       │       ├── cine_sax_info.csv
│       │       ├── cine_sax_ks.mat
│       │       ├── t1map_calib.mat
│       │       ├── t1map_info.csv
│       │       ├── t1map_ks.mat
│       │       ├── t2map_calib.mat
│       │       ├── t2map_info.csv
│       │       └── t2map_ks.mat
│       ├── P032
│       │       ├── cine_lax_calib.mat
│       │       ├── cine_lax_info.csv
│       │       ├── cine_lax_ks.mat
│       │       ├── cine_sax_calib.mat
│       │       ├── cine_sax_info.csv
│       │       ├── cine_sax_ks.mat
│       │       ├── t1map_calib.mat
│       │       ├── t1map_info.csv
```

```
│       │    ├── t1map_ks.mat
│       │    ├── t2map_calib.mat
│       │    ├── t2map_info.csv
│       │    └── t2map_ks.mat
│       ├── P033
│       │    ├── cine_lax_calib.mat
│       │    ├── cine_lax_info.csv
│       │    ├── cine_lax_ks.mat
│       │    ├── cine_sax_calib.mat
│       │    ├── cine_sax_info.csv
│       │    ├── cine_sax_ks.mat
│       │    ├── t1map_calib.mat
│       │    ├── t1map_info.csv
│       │    ├── t1map_ks.mat
│       │    ├── t2map_calib.mat
│       │    ├── t2map_info.csv
│       │    └── t2map_ks.mat
│       ├── P034
│       │    ├── cine_lax_calib.mat
│       │    ├── cine_lax_info.csv
│       │    ├── cine_lax_ks.mat
│       │    ├── cine_sax_calib.mat
│       │    ├── cine_sax_info.csv
│       │    ├── cine_sax_ks.mat
│       │    ├── t1map_calib.mat
│       │    ├── t1map_info.csv
│       │    ├── t1map_ks.mat
│       │    ├── t2map_calib.mat
│       │    ├── t2map_info.csv
│       │    └── t2map_ks.mat
│       ├── P035
│       │    ├── cine_lax_calib.mat
│       │    ├── cine_lax_info.csv
│       │    ├── cine_lax_ks.mat
│       │    ├── cine_sax_calib.mat
│       │    ├── cine_sax_info.csv
│       │    ├── cine_sax_ks.mat
│       │    ├── t1map_calib.mat
│       │    ├── t1map_info.csv
│       │    ├── t1map_ks.mat
│       │    ├── t2map_calib.mat
│       │    ├── t2map_info.csv
│       │    └── t2map_ks.mat
│       ├── P036
│       │    ├── cine_lax_calib.mat
│       │    ├── cine_lax_info.csv
│       │    ├── cine_lax_ks.mat
│       │    ├── cine_sax_calib.mat
│       │    ├── cine_sax_info.csv
│       │    ├── cine_sax_ks.mat
│       │    ├── t1map_calib.mat
│       │    ├── t1map_info.csv
│       │    ├── t1map_ks.mat
│       │    ├── t2map_calib.mat
```

```
│       │   ├── t2map_info.csv
│       │   └── t2map_ks.mat
│       ├── P037
│       │   ├── cine_lax_calib.mat
│       │   ├── cine_lax_info.csv
│       │   ├── cine_lax_ks.mat
│       │   ├── cine_sax_calib.mat
│       │   ├── cine_sax_info.csv
│       │   ├── cine_sax_ks.mat
│       │   ├── t1map_calib.mat
│       │   ├── t1map_info.csv
│       │   ├── t1map_ks.mat
│       │   ├── t2map_calib.mat
│       │   ├── t2map_info.csv
│       │   └── t2map_ks.mat
│       ├── P038
│       │   ├── cine_lax_calib.mat
│       │   ├── cine_lax_info.csv
│       │   ├── cine_lax_ks.mat
│       │   ├── cine_sax_calib.mat
│       │   ├── cine_sax_info.csv
│       │   ├── cine_sax_ks.mat
│       │   ├── t1map_calib.mat
│       │   ├── t1map_info.csv
│       │   ├── t1map_ks.mat
│       │   ├── t2map_calib.mat
│       │   ├── t2map_info.csv
│       │   └── t2map_ks.mat
│       ├── P039
│       │   ├── cine_lax_calib.mat
│       │   ├── cine_lax_info.csv
│       │   ├── cine_lax_ks.mat
│       │   ├── cine_sax_calib.mat
│       │   ├── cine_sax_info.csv
│       │   ├── cine_sax_ks.mat
│       │   ├── t1map_calib.mat
│       │   ├── t1map_info.csv
│       │   ├── t1map_ks.mat
│       │   ├── t2map_calib.mat
│       │   ├── t2map_info.csv
│       │   └── t2map_ks.mat
│       ├── P040
│       │   ├── cine_lax_calib.mat
│       │   ├── cine_lax_info.csv
│       │   ├── cine_lax_ks.mat
│       │   ├── cine_sax_calib.mat
│       │   ├── cine_sax_info.csv
│       │   ├── cine_sax_ks.mat
│       │   ├── t1map_calib.mat
│       │   ├── t1map_info.csv
│       │   ├── t1map_ks.mat
│       │   ├── t2map_calib.mat
│       │   ├── t2map_info.csv
│       │   └── t2map_ks.mat
```

```
│   ├── P041
│   │   ├── cine_lax_calib.mat
│   │   ├── cine_lax_info.csv
│   │   ├── cine_lax_ks.mat
│   │   ├── cine_sax_calib.mat
│   │   ├── cine_sax_info.csv
│   │   ├── cine_sax_ks.mat
│   │   ├── t1map_calib.mat
│   │   ├── t1map_info.csv
│   │   ├── t1map_ks.mat
│   │   ├── t2map_calib.mat
│   │   ├── t2map_info.csv
│   │   └── t2map_ks.mat
│   ├── P042
│   │   ├── cine_lax_calib.mat
│   │   ├── cine_lax_info.csv
│   │   ├── cine_lax_ks.mat
│   │   ├── cine_sax_calib.mat
│   │   ├── cine_sax_info.csv
│   │   ├── cine_sax_ks.mat
│   │   ├── t1map_calib.mat
│   │   ├── t1map_info.csv
│   │   ├── t1map_ks.mat
│   │   ├── t2map_calib.mat
│   │   ├── t2map_info.csv
│   │   └── t2map_ks.mat
│   ├── P043
│   │   ├── cine_lax_calib.mat
│   │   ├── cine_lax_info.csv
│   │   ├── cine_lax_ks.mat
│   │   ├── cine_sax_calib.mat
│   │   ├── cine_sax_info.csv
│   │   ├── cine_sax_ks.mat
│   │   ├── t1map_calib.mat
│   │   ├── t1map_info.csv
│   │   ├── t1map_ks.mat
│   │   ├── t2map_calib.mat
│   │   ├── t2map_info.csv
│   │   └── t2map_ks.mat
│   ├── P044
│   │   ├── cine_lax_calib.mat
│   │   ├── cine_lax_info.csv
│   │   ├── cine_lax_ks.mat
│   │   ├── cine_sax_calib.mat
│   │   ├── cine_sax_info.csv
│   │   ├── cine_sax_ks.mat
│   │   ├── t1map_calib.mat
│   │   ├── t1map_info.csv
│   │   ├── t1map_ks.mat
│   │   ├── t2map_calib.mat
│   │   ├── t2map_info.csv
│   │   └── t2map_ks.mat
│   ├── P045
│   │   ├── cine_lax_calib.mat
```

```
│       │       ├── cine_lax_info.csv
│       │       ├── cine_lax_ks.mat
│       │       ├── cine_sax_calib.mat
│       │       ├── cine_sax_info.csv
│       │       ├── cine_sax_ks.mat
│       │       ├── t1map_calib.mat
│       │       ├── t1map_info.csv
│       │       ├── t1map_ks.mat
│       │       ├── t2map_calib.mat
│       │       ├── t2map_info.csv
│       │       └── t2map_ks.mat
│       ├── P046
│       │       ├── cine_lax_calib.mat
│       │       ├── cine_lax_info.csv
│       │       ├── cine_lax_ks.mat
│       │       ├── cine_sax_calib.mat
│       │       ├── cine_sax_info.csv
│       │       ├── cine_sax_ks.mat
│       │       ├── t1map_calib.mat
│       │       ├── t1map_info.csv
│       │       ├── t1map_ks.mat
│       │       ├── t2map_calib.mat
│       │       ├── t2map_info.csv
│       │       └── t2map_ks.mat
│       ├── P047
│       │       ├── cine_lax_calib.mat
│       │       ├── cine_lax_info.csv
│       │       ├── cine_lax_ks.mat
│       │       ├── cine_sax_calib.mat
│       │       ├── cine_sax_info.csv
│       │       ├── cine_sax_ks.mat
│       │       ├── t1map_calib.mat
│       │       ├── t1map_info.csv
│       │       ├── t1map_ks.mat
│       │       ├── t2map_calib.mat
│       │       ├── t2map_info.csv
│       │       └── t2map_ks.mat
│       ├── P048
│       │       ├── cine_lax_calib.mat
│       │       ├── cine_lax_info.csv
│       │       ├── cine_lax_ks.mat
│       │       ├── cine_sax_calib.mat
│       │       ├── cine_sax_info.csv
│       │       ├── cine_sax_ks.mat
│       │       ├── t1map_calib.mat
│       │       ├── t1map_info.csv
│       │       ├── t1map_ks.mat
│       │       ├── t2map_calib.mat
│       │       ├── t2map_info.csv
│       │       └── t2map_ks.mat
│       ├── P049
│       │       ├── cine_lax_calib.mat
│       │       ├── cine_lax_info.csv
│       │       ├── cine_lax_ks.mat
```

```
│       │       ├── cine_sax_calib.mat
│       │       ├── cine_sax_info.csv
│       │       ├── cine_sax_ks.mat
│       │       ├── t1map_calib.mat
│       │       ├── t1map_info.csv
│       │       ├── t1map_ks.mat
│       │       ├── t2map_calib.mat
│       │       ├── t2map_info.csv
│       │       └── t2map_ks.mat
│       ├── P050
│       │       ├── cine_lax_calib.mat
│       │       ├── cine_lax_info.csv
│       │       ├── cine_lax_ks.mat
│       │       ├── cine_sax_calib.mat
│       │       ├── cine_sax_info.csv
│       │       ├── cine_sax_ks.mat
│       │       ├── t1map_calib.mat
│       │       ├── t1map_info.csv
│       │       ├── t1map_ks.mat
│       │       ├── t2map_calib.mat
│       │       ├── t2map_info.csv
│       │       └── t2map_ks.mat
│       ├── P051
│       │       ├── cine_lax_calib.mat
│       │       ├── cine_lax_info.csv
│       │       ├── cine_lax_ks.mat
│       │       ├── cine_sax_calib.mat
│       │       ├── cine_sax_info.csv
│       │       ├── cine_sax_ks.mat
│       │       ├── t1map_calib.mat
│       │       ├── t1map_info.csv
│       │       ├── t1map_ks.mat
│       │       ├── t2map_calib.mat
│       │       ├── t2map_info.csv
│       │       └── t2map_ks.mat
│       ├── P052
│       │       ├── cine_lax_calib.mat
│       │       ├── cine_lax_info.csv
│       │       ├── cine_lax_ks.mat
│       │       ├── cine_sax_calib.mat
│       │       ├── cine_sax_info.csv
│       │       ├── cine_sax_ks.mat
│       │       ├── t1map_calib.mat
│       │       ├── t1map_info.csv
│       │       ├── t1map_ks.mat
│       │       ├── t2map_calib.mat
│       │       ├── t2map_info.csv
│       │       └── t2map_ks.mat
│       ├── P053
│       │       ├── cine_lax_calib.mat
│       │       ├── cine_lax_info.csv
│       │       ├── cine_lax_ks.mat
│       │       ├── cine_sax_calib.mat
│       │       ├── cine_sax_info.csv
```

```
│       │       ├── cine_sax_ks.mat
│       │       ├── t1map_calib.mat
│       │       ├── t1map_info.csv
│       │       ├── t1map_ks.mat
│       │       ├── t2map_calib.mat
│       │       ├── t2map_info.csv
│       │       └── t2map_ks.mat
│       ├── P054
│       │       ├── cine_lax_calib.mat
│       │       ├── cine_lax_info.csv
│       │       ├── cine_lax_ks.mat
│       │       ├── cine_sax_calib.mat
│       │       ├── cine_sax_info.csv
│       │       ├── cine_sax_ks.mat
│       │       ├── t1map_calib.mat
│       │       ├── t1map_info.csv
│       │       ├── t1map_ks.mat
│       │       ├── t2map_calib.mat
│       │       ├── t2map_info.csv
│       │       └── t2map_ks.mat
│       ├── P055
│       │       ├── cine_lax_calib.mat
│       │       ├── cine_lax_info.csv
│       │       ├── cine_lax_ks.mat
│       │       ├── cine_sax_calib.mat
│       │       ├── cine_sax_info.csv
│       │       ├── cine_sax_ks.mat
│       │       ├── t1map_calib.mat
│       │       ├── t1map_info.csv
│       │       ├── t1map_ks.mat
│       │       ├── t2map_calib.mat
│       │       ├── t2map_info.csv
│       │       └── t2map_ks.mat
│       ├── P056
│       │       ├── cine_lax_calib.mat
│       │       ├── cine_lax_info.csv
│       │       ├── cine_lax_ks.mat
│       │       ├── cine_sax_calib.mat
│       │       ├── cine_sax_info.csv
│       │       ├── cine_sax_ks.mat
│       │       ├── t1map_calib.mat
│       │       ├── t1map_info.csv
│       │       ├── t1map_ks.mat
│       │       ├── t2map_calib.mat
│       │       ├── t2map_info.csv
│       │       └── t2map_ks.mat
│       ├── P057
│       │       ├── cine_lax_calib.mat
│       │       ├── cine_lax_info.csv
│       │       ├── cine_lax_ks.mat
│       │       ├── cine_sax_calib.mat
│       │       ├── cine_sax_info.csv
│       │       ├── cine_sax_ks.mat
│       │       ├── t1map_calib.mat
```

```
│       │       ├── t1map_info.csv
│       │       ├── t1map_ks.mat
│       │       ├── t2map_calib.mat
│       │       ├── t2map_info.csv
│       │       └── t2map_ks.mat
│       ├── P058
│       │       ├── cine_lax_calib.mat
│       │       ├── cine_lax_info.csv
│       │       ├── cine_lax_ks.mat
│       │       ├── cine_sax_calib.mat
│       │       ├── cine_sax_info.csv
│       │       ├── cine_sax_ks.mat
│       │       ├── t1map_calib.mat
│       │       ├── t1map_info.csv
│       │       ├── t1map_ks.mat
│       │       ├── t2map_calib.mat
│       │       ├── t2map_info.csv
│       │       └── t2map_ks.mat
│       ├── P059
│       │       ├── cine_lax_calib.mat
│       │       ├── cine_lax_info.csv
│       │       ├── cine_lax_ks.mat
│       │       ├── cine_sax_calib.mat
│       │       ├── cine_sax_info.csv
│       │       ├── cine_sax_ks.mat
│       │       ├── t1map_calib.mat
│       │       ├── t1map_info.csv
│       │       ├── t1map_ks.mat
│       │       ├── t2map_calib.mat
│       │       ├── t2map_info.csv
│       │       └── t2map_ks.mat
│       ├── P060
│       │       ├── cine_lax_calib.mat
│       │       ├── cine_lax_info.csv
│       │       ├── cine_lax_ks.mat
│       │       ├── cine_sax_calib.mat
│       │       ├── cine_sax_info.csv
│       │       ├── cine_sax_ks.mat
│       │       ├── t1map_calib.mat
│       │       ├── t1map_info.csv
│       │       ├── t1map_ks.mat
│       │       ├── t2map_calib.mat
│       │       ├── t2map_info.csv
│       │       └── t2map_ks.mat
│       ├── P061
│       │       ├── cine_lax_calib.mat
│       │       ├── cine_lax_info.csv
│       │       ├── cine_lax_ks.mat
│       │       ├── cine_sax_calib.mat
│       │       ├── cine_sax_info.csv
│       │       ├── cine_sax_ks.mat
│       │       ├── t1map_calib.mat
│       │       ├── t1map_info.csv
│       │       ├── t1map_ks.mat
```

```
│       │       ├── t2map_calib.mat
│       │       ├── t2map_info.csv
│       │       └── t2map_ks.mat
│       ├── P062
│       │       ├── cine_lax_calib.mat
│       │       ├── cine_lax_info.csv
│       │       ├── cine_lax_ks.mat
│       │       ├── cine_sax_calib.mat
│       │       ├── cine_sax_info.csv
│       │       ├── cine_sax_ks.mat
│       │       ├── t1map_calib.mat
│       │       ├── t1map_info.csv
│       │       ├── t1map_ks.mat
│       │       ├── t2map_calib.mat
│       │       ├── t2map_info.csv
│       │       └── t2map_ks.mat
│       ├── P063
│       │       ├── cine_lax_calib.mat
│       │       ├── cine_lax_info.csv
│       │       ├── cine_lax_ks.mat
│       │       ├── cine_sax_calib.mat
│       │       ├── cine_sax_info.csv
│       │       ├── cine_sax_ks.mat
│       │       ├── t1map_calib.mat
│       │       ├── t1map_info.csv
│       │       ├── t1map_ks.mat
│       │       ├── t2map_calib.mat
│       │       ├── t2map_info.csv
│       │       └── t2map_ks.mat
│       ├── P064
│       │       ├── cine_lax_calib.mat
│       │       ├── cine_lax_info.csv
│       │       ├── cine_lax_ks.mat
│       │       ├── cine_sax_calib.mat
│       │       ├── cine_sax_info.csv
│       │       ├── cine_sax_ks.mat
│       │       ├── t1map_calib.mat
│       │       ├── t1map_info.csv
│       │       ├── t1map_ks.mat
│       │       ├── t2map_calib.mat
│       │       ├── t2map_info.csv
│       │       └── t2map_ks.mat
│       ├── P065
│       │       ├── cine_lax_calib.mat
│       │       ├── cine_lax_info.csv
│       │       ├── cine_lax_ks.mat
│       │       ├── cine_sax_calib.mat
│       │       ├── cine_sax_info.csv
│       │       ├── cine_sax_ks.mat
│       │       ├── t1map_calib.mat
│       │       ├── t1map_info.csv
│       │       ├── t1map_ks.mat
│       │       ├── t2map_calib.mat
│       │       ├── t2map_info.csv
```

```
│       │   └── t2map_ks.mat
│       ├── P066
│       │   ├── cine_lax_calib.mat
│       │   ├── cine_lax_info.csv
│       │   ├── cine_lax_ks.mat
│       │   ├── cine_sax_calib.mat
│       │   ├── cine_sax_info.csv
│       │   ├── cine_sax_ks.mat
│       │   ├── t1map_calib.mat
│       │   ├── t1map_info.csv
│       │   ├── t1map_ks.mat
│       │   ├── t2map_calib.mat
│       │   ├── t2map_info.csv
│       │   └── t2map_ks.mat
│       ├── P067
│       │   ├── cine_lax_calib.mat
│       │   ├── cine_lax_info.csv
│       │   ├── cine_lax_ks.mat
│       │   ├── cine_sax_calib.mat
│       │   ├── cine_sax_info.csv
│       │   ├── cine_sax_ks.mat
│       │   ├── t1map_calib.mat
│       │   ├── t1map_info.csv
│       │   ├── t1map_ks.mat
│       │   ├── t2map_calib.mat
│       │   ├── t2map_info.csv
│       │   └── t2map_ks.mat
│       ├── P068
│       │   ├── cine_lax_calib.mat
│       │   ├── cine_lax_info.csv
│       │   ├── cine_lax_ks.mat
│       │   ├── cine_sax_calib.mat
│       │   ├── cine_sax_info.csv
│       │   ├── cine_sax_ks.mat
│       │   ├── t1map_calib.mat
│       │   ├── t1map_info.csv
│       │   ├── t1map_ks.mat
│       │   ├── t2map_calib.mat
│       │   ├── t2map_info.csv
│       │   └── t2map_ks.mat
│       ├── P069
│       │   ├── cine_lax_calib.mat
│       │   ├── cine_lax_info.csv
│       │   ├── cine_lax_ks.mat
│       │   ├── cine_sax_calib.mat
│       │   ├── cine_sax_info.csv
│       │   ├── cine_sax_ks.mat
│       │   ├── t1map_calib.mat
│       │   ├── t1map_info.csv
│       │   ├── t1map_ks.mat
│       │   ├── t2map_calib.mat
│       │   ├── t2map_info.csv
│       │   └── t2map_ks.mat
│       ├── P070
```

```
│       │       ├── cine_lax_calib.mat
│       │       ├── cine_lax_info.csv
│       │       ├── cine_lax_ks.mat
│       │       ├── cine_sax_calib.mat
│       │       ├── cine_sax_info.csv
│       │       ├── cine_sax_ks.mat
│       │       ├── t1map_calib.mat
│       │       ├── t1map_info.csv
│       │       ├── t1map_ks.mat
│       │       ├── t2map_calib.mat
│       │       ├── t2map_info.csv
│       │       └── t2map_ks.mat
│       ├── P071
│       │       ├── cine_lax_calib.mat
│       │       ├── cine_lax_info.csv
│       │       ├── cine_lax_ks.mat
│       │       ├── cine_sax_calib.mat
│       │       ├── cine_sax_info.csv
│       │       ├── cine_sax_ks.mat
│       │       ├── t1map_calib.mat
│       │       ├── t1map_info.csv
│       │       ├── t1map_ks.mat
│       │       ├── t2map_calib.mat
│       │       ├── t2map_info.csv
│       │       └── t2map_ks.mat
│       ├── P072
│       │       ├── cine_lax_calib.mat
│       │       ├── cine_lax_info.csv
│       │       ├── cine_lax_ks.mat
│       │       ├── cine_sax_calib.mat
│       │       ├── cine_sax_info.csv
│       │       ├── cine_sax_ks.mat
│       │       ├── t1map_calib.mat
│       │       ├── t1map_info.csv
│       │       ├── t1map_ks.mat
│       │       ├── t2map_calib.mat
│       │       ├── t2map_info.csv
│       │       └── t2map_ks.mat
│       ├── P073
│       │       ├── cine_lax_calib.mat
│       │       ├── cine_lax_info.csv
│       │       ├── cine_lax_ks.mat
│       │       ├── cine_sax_calib.mat
│       │       ├── cine_sax_info.csv
│       │       ├── cine_sax_ks.mat
│       │       ├── t1map_calib.mat
│       │       ├── t1map_info.csv
│       │       ├── t1map_ks.mat
│       │       ├── t2map_calib.mat
│       │       ├── t2map_info.csv
│       │       └── t2map_ks.mat
│       ├── P074
│       │       ├── cine_lax_calib.mat
│       │       ├── cine_lax_info.csv
```

```
│       │     ├── cine_lax_ks.mat
│       │     ├── cine_sax_calib.mat
│       │     ├── cine_sax_info.csv
│       │     ├── cine_sax_ks.mat
│       │     ├── t1map_calib.mat
│       │     ├── t1map_info.csv
│       │     ├── t1map_ks.mat
│       │     ├── t2map_calib.mat
│       │     ├── t2map_info.csv
│       │     └── t2map_ks.mat
│       ├── P075
│       │     ├── cine_lax_calib.mat
│       │     ├── cine_lax_info.csv
│       │     ├── cine_lax_ks.mat
│       │     ├── cine_sax_calib.mat
│       │     ├── cine_sax_info.csv
│       │     ├── cine_sax_ks.mat
│       │     ├── t1map_calib.mat
│       │     ├── t1map_info.csv
│       │     ├── t1map_ks.mat
│       │     ├── t2map_calib.mat
│       │     ├── t2map_info.csv
│       │     └── t2map_ks.mat
│       ├── P076
│       │     ├── cine_lax_calib.mat
│       │     ├── cine_lax_info.csv
│       │     ├── cine_lax_ks.mat
│       │     ├── cine_sax_calib.mat
│       │     ├── cine_sax_info.csv
│       │     ├── cine_sax_ks.mat
│       │     ├── t1map_calib.mat
│       │     ├── t1map_info.csv
│       │     ├── t1map_ks.mat
│       │     ├── t2map_calib.mat
│       │     ├── t2map_info.csv
│       │     └── t2map_ks.mat
│       ├── P077
│       │     ├── cine_lax_calib.mat
│       │     ├── cine_lax_info.csv
│       │     ├── cine_lax_ks.mat
│       │     ├── cine_sax_calib.mat
│       │     ├── cine_sax_info.csv
│       │     ├── cine_sax_ks.mat
│       │     ├── t1map_calib.mat
│       │     ├── t1map_info.csv
│       │     ├── t1map_ks.mat
│       │     ├── t2map_calib.mat
│       │     ├── t2map_info.csv
│       │     └── t2map_ks.mat
│       ├── P078
│       │     ├── cine_lax_calib.mat
│       │     ├── cine_lax_info.csv
│       │     ├── cine_lax_ks.mat
│       │     ├── cine_sax_calib.mat
```

```
│       │       ├── cine_sax_info.csv
│       │       ├── cine_sax_ks.mat
│       │       ├── t1map_calib.mat
│       │       ├── t1map_info.csv
│       │       ├── t1map_ks.mat
│       │       ├── t2map_calib.mat
│       │       ├── t2map_info.csv
│       │       └── t2map_ks.mat
│       ├── P079
│       │       ├── cine_lax_calib.mat
│       │       ├── cine_lax_info.csv
│       │       ├── cine_lax_ks.mat
│       │       ├── cine_sax_calib.mat
│       │       ├── cine_sax_info.csv
│       │       ├── cine_sax_ks.mat
│       │       ├── t1map_calib.mat
│       │       ├── t1map_info.csv
│       │       ├── t1map_ks.mat
│       │       ├── t2map_calib.mat
│       │       ├── t2map_info.csv
│       │       └── t2map_ks.mat
│       ├── P080
│       │       ├── cine_lax_calib.mat
│       │       ├── cine_lax_info.csv
│       │       ├── cine_lax_ks.mat
│       │       ├── t1map_calib.mat
│       │       ├── t1map_info.csv
│       │       ├── t1map_ks.mat
│       │       ├── t2map_calib.mat
│       │       ├── t2map_info.csv
│       │       └── t2map_ks.mat
│       ├── P081
│       │       ├── cine_lax_calib.mat
│       │       ├── cine_lax_info.csv
│       │       ├── cine_lax_ks.mat
│       │       ├── cine_sax_calib.mat
│       │       ├── cine_sax_info.csv
│       │       ├── cine_sax_ks.mat
│       │       ├── t1map_calib.mat
│       │       ├── t1map_info.csv
│       │       ├── t1map_ks.mat
│       │       ├── t2map_calib.mat
│       │       ├── t2map_info.csv
│       │       └── t2map_ks.mat
│       ├── P082
│       │       ├── cine_lax_calib.mat
│       │       ├── cine_lax_info.csv
│       │       ├── cine_lax_ks.mat
│       │       ├── cine_sax_calib.mat
│       │       ├── cine_sax_info.csv
│       │       ├── cine_sax_ks.mat
│       │       ├── t1map_calib.mat
│       │       ├── t1map_info.csv
│       │       ├── t1map_ks.mat
```

```
│       │       ├── t2map_calib.mat
│       │       ├── t2map_info.csv
│       │       └── t2map_ks.mat
│       ├── P083
│       │       ├── cine_lax_calib.mat
│       │       ├── cine_lax_info.csv
│       │       ├── cine_lax_ks.mat
│       │       ├── cine_sax_calib.mat
│       │       ├── cine_sax_info.csv
│       │       ├── cine_sax_ks.mat
│       │       ├── t1map_calib.mat
│       │       ├── t1map_info.csv
│       │       ├── t1map_ks.mat
│       │       ├── t2map_calib.mat
│       │       ├── t2map_info.csv
│       │       └── t2map_ks.mat
│       ├── P084
│       │       ├── cine_lax_calib.mat
│       │       ├── cine_lax_info.csv
│       │       ├── cine_lax_ks.mat
│       │       ├── cine_sax_calib.mat
│       │       ├── cine_sax_info.csv
│       │       ├── cine_sax_ks.mat
│       │       ├── t1map_calib.mat
│       │       ├── t1map_info.csv
│       │       ├── t1map_ks.mat
│       │       ├── t2map_calib.mat
│       │       ├── t2map_info.csv
│       │       └── t2map_ks.mat
│       ├── P085
│       │       ├── cine_lax_calib.mat
│       │       ├── cine_lax_info.csv
│       │       ├── cine_lax_ks.mat
│       │       ├── cine_sax_calib.mat
│       │       ├── cine_sax_info.csv
│       │       ├── cine_sax_ks.mat
│       │       ├── t1map_calib.mat
│       │       ├── t1map_info.csv
│       │       ├── t1map_ks.mat
│       │       ├── t2map_calib.mat
│       │       ├── t2map_info.csv
│       │       └── t2map_ks.mat
│       ├── P086
│       │       ├── cine_lax_calib.mat
│       │       ├── cine_lax_info.csv
│       │       ├── cine_lax_ks.mat
│       │       ├── cine_sax_calib.mat
│       │       ├── cine_sax_info.csv
│       │       ├── cine_sax_ks.mat
│       │       ├── t1map_calib.mat
│       │       ├── t1map_info.csv
│       │       ├── t1map_ks.mat
│       │       ├── t2map_calib.mat
│       │       ├── t2map_info.csv
```

```
│       │       └── t2map_ks.mat
│       ├── P087
│       │       ├── cine_lax_calib.mat
│       │       ├── cine_lax_info.csv
│       │       ├── cine_lax_ks.mat
│       │       ├── cine_sax_calib.mat
│       │       ├── cine_sax_info.csv
│       │       ├── cine_sax_ks.mat
│       │       ├── t1map_calib.mat
│       │       ├── t1map_info.csv
│       │       ├── t1map_ks.mat
│       │       ├── t2map_calib.mat
│       │       ├── t2map_info.csv
│       │       └── t2map_ks.mat
│       ├── P088
│       │       ├── cine_lax_calib.mat
│       │       ├── cine_lax_info.csv
│       │       ├── cine_lax_ks.mat
│       │       ├── cine_sax_calib.mat
│       │       ├── cine_sax_info.csv
│       │       ├── cine_sax_ks.mat
│       │       ├── t1map_calib.mat
│       │       ├── t1map_info.csv
│       │       ├── t1map_ks.mat
│       │       ├── t2map_calib.mat
│       │       ├── t2map_info.csv
│       │       └── t2map_ks.mat
│       ├── P089
│       │       ├── cine_lax_calib.mat
│       │       ├── cine_lax_info.csv
│       │       ├── cine_lax_ks.mat
│       │       ├── cine_sax_calib.mat
│       │       ├── cine_sax_info.csv
│       │       ├── cine_sax_ks.mat
│       │       ├── t1map_calib.mat
│       │       ├── t1map_info.csv
│       │       ├── t1map_ks.mat
│       │       ├── t2map_calib.mat
│       │       ├── t2map_info.csv
│       │       └── t2map_ks.mat
│       ├── P090
│       │       ├── cine_lax_calib.mat
│       │       ├── cine_lax_info.csv
│       │       ├── cine_lax_ks.mat
│       │       ├── cine_sax_calib.mat
│       │       ├── cine_sax_info.csv
│       │       ├── cine_sax_ks.mat
│       │       ├── t1map_calib.mat
│       │       ├── t1map_info.csv
│       │       ├── t1map_ks.mat
│       │       ├── t2map_calib.mat
│       │       ├── t2map_info.csv
│       │       └── t2map_ks.mat
│       ├── P091
```

```
│       │       ├── cine_lax_calib.mat
│       │       ├── cine_lax_info.csv
│       │       ├── cine_lax_ks.mat
│       │       ├── cine_sax_calib.mat
│       │       ├── cine_sax_info.csv
│       │       ├── cine_sax_ks.mat
│       │       ├── t1map_calib.mat
│       │       ├── t1map_info.csv
│       │       ├── t1map_ks.mat
│       │       ├── t2map_calib.mat
│       │       ├── t2map_info.csv
│       │       └── t2map_ks.mat
│       ├── P092
│       │       ├── cine_lax_calib.mat
│       │       ├── cine_lax_info.csv
│       │       ├── cine_lax_ks.mat
│       │       ├── cine_sax_calib.mat
│       │       ├── cine_sax_info.csv
│       │       ├── cine_sax_ks.mat
│       │       ├── t1map_calib.mat
│       │       ├── t1map_info.csv
│       │       ├── t1map_ks.mat
│       │       ├── t2map_calib.mat
│       │       ├── t2map_info.csv
│       │       └── t2map_ks.mat
│       ├── P093
│       │       ├── cine_lax_calib.mat
│       │       ├── cine_lax_info.csv
│       │       ├── cine_lax_ks.mat
│       │       ├── cine_sax_calib.mat
│       │       ├── cine_sax_info.csv
│       │       ├── cine_sax_ks.mat
│       │       ├── t1map_calib.mat
│       │       ├── t1map_info.csv
│       │       ├── t1map_ks.mat
│       │       ├── t2map_calib.mat
│       │       ├── t2map_info.csv
│       │       └── t2map_ks.mat
│       ├── P094
│       │       ├── cine_lax_calib.mat
│       │       ├── cine_lax_info.csv
│       │       ├── cine_lax_ks.mat
│       │       ├── cine_sax_calib.mat
│       │       ├── cine_sax_info.csv
│       │       ├── cine_sax_ks.mat
│       │       ├── t1map_calib.mat
│       │       ├── t1map_info.csv
│       │       ├── t1map_ks.mat
│       │       ├── t2map_calib.mat
│       │       ├── t2map_info.csv
│       │       └── t2map_ks.mat
│       ├── P095
│       │       ├── cine_lax_calib.mat
│       │       ├── cine_lax_info.csv
```

```
│       │       ├── cine_lax_ks.mat
│       │       ├── cine_sax_calib.mat
│       │       ├── cine_sax_info.csv
│       │       ├── cine_sax_ks.mat
│       │       ├── t1map_calib.mat
│       │       ├── t1map_info.csv
│       │       ├── t1map_ks.mat
│       │       ├── t2map_calib.mat
│       │       ├── t2map_info.csv
│       │       └── t2map_ks.mat
│       ├── P096
│       │       ├── cine_lax_calib.mat
│       │       ├── cine_lax_info.csv
│       │       ├── cine_lax_ks.mat
│       │       ├── cine_sax_calib.mat
│       │       ├── cine_sax_info.csv
│       │       ├── cine_sax_ks.mat
│       │       ├── t1map_calib.mat
│       │       ├── t1map_info.csv
│       │       ├── t1map_ks.mat
│       │       ├── t2map_calib.mat
│       │       ├── t2map_info.csv
│       │       └── t2map_ks.mat
│       ├── P097
│       │       ├── cine_lax_calib.mat
│       │       ├── cine_lax_info.csv
│       │       ├── cine_lax_ks.mat
│       │       ├── cine_sax_calib.mat
│       │       ├── cine_sax_info.csv
│       │       ├── cine_sax_ks.mat
│       │       ├── t1map_calib.mat
│       │       ├── t1map_info.csv
│       │       ├── t1map_ks.mat
│       │       ├── t2map_calib.mat
│       │       ├── t2map_info.csv
│       │       └── t2map_ks.mat
│       ├── P098
│       │       ├── cine_lax_calib.mat
│       │       ├── cine_lax_info.csv
│       │       ├── cine_lax_ks.mat
│       │       ├── cine_sax_calib.mat
│       │       ├── cine_sax_info.csv
│       │       ├── cine_sax_ks.mat
│       │       ├── t1map_calib.mat
│       │       ├── t1map_info.csv
│       │       ├── t1map_ks.mat
│       │       ├── t2map_calib.mat
│       │       ├── t2map_info.csv
│       │       └── t2map_ks.mat
│       ├── P099
│       │       ├── cine_lax_calib.mat
│       │       ├── cine_lax_info.csv
│       │       ├── cine_lax_ks.mat
│       │       ├── cine_sax_calib.mat
```

```
│       │       ├── cine_sax_info.csv
│       │       ├── cine_sax_ks.mat
│       │       ├── t1map_calib.mat
│       │       ├── t1map_info.csv
│       │       ├── t1map_ks.mat
│       │       ├── t2map_calib.mat
│       │       ├── t2map_info.csv
│       │       └── t2map_ks.mat
│       ├── P100
│       │   ├── cine_lax_calib.mat
│       │   ├── cine_lax_info.csv
│       │   ├── cine_lax_ks.mat
│       │   ├── cine_sax_calib.mat
│       │   ├── cine_sax_info.csv
│       │   ├── cine_sax_ks.mat
│       │   ├── t1map_calib.mat
│       │   ├── t1map_info.csv
│       │   ├── t1map_ks.mat
│       │   ├── t2map_calib.mat
│       │   ├── t2map_info.csv
│       │   └── t2map_ks.mat
│       ├── P101
│       │   ├── cine_lax_calib.mat
│       │   ├── cine_lax_info.csv
│       │   ├── cine_lax_ks.mat
│       │   ├── cine_sax_calib.mat
│       │   ├── cine_sax_info.csv
│       │   ├── cine_sax_ks.mat
│       │   ├── t1map_calib.mat
│       │   ├── t1map_info.csv
│       │   ├── t1map_ks.mat
│       │   ├── t2map_calib.mat
│       │   ├── t2map_info.csv
│       │   └── t2map_ks.mat
│       ├── P102
│       │   ├── cine_lax_calib.mat
│       │   ├── cine_lax_info.csv
│       │   ├── cine_lax_ks.mat
│       │   ├── cine_sax_calib.mat
│       │   ├── cine_sax_info.csv
│       │   ├── cine_sax_ks.mat
│       │   ├── t1map_calib.mat
│       │   ├── t1map_info.csv
│       │   ├── t1map_ks.mat
│       │   ├── t2map_calib.mat
│       │   ├── t2map_info.csv
│       │   └── t2map_ks.mat
│       ├── P103
│       │   ├── cine_lax_calib.mat
│       │   ├── cine_lax_info.csv
│       │   ├── cine_lax_ks.mat
│       │   ├── cine_sax_calib.mat
│       │   ├── cine_sax_info.csv
│       │   ├── cine_sax_ks.mat
```

```
│       │       ├── t1map_calib.mat
│       │       ├── t1map_info.csv
│       │       ├── t1map_ks.mat
│       │       ├── t2map_calib.mat
│       │       ├── t2map_info.csv
│       │       └── t2map_ks.mat
│       ├── P104
│       │       ├── cine_lax_calib.mat
│       │       ├── cine_lax_info.csv
│       │       ├── cine_lax_ks.mat
│       │       ├── cine_sax_calib.mat
│       │       ├── cine_sax_info.csv
│       │       ├── cine_sax_ks.mat
│       │       ├── t1map_calib.mat
│       │       ├── t1map_info.csv
│       │       ├── t1map_ks.mat
│       │       ├── t2map_calib.mat
│       │       ├── t2map_info.csv
│       │       └── t2map_ks.mat
│       ├── P105
│       │       ├── cine_lax_calib.mat
│       │       ├── cine_lax_info.csv
│       │       ├── cine_lax_ks.mat
│       │       ├── cine_sax_calib.mat
│       │       ├── cine_sax_info.csv
│       │       ├── cine_sax_ks.mat
│       │       ├── t1map_calib.mat
│       │       ├── t1map_info.csv
│       │       ├── t1map_ks.mat
│       │       ├── t2map_calib.mat
│       │       ├── t2map_info.csv
│       │       └── t2map_ks.mat
│       ├── P106
│       │       ├── cine_lax_calib.mat
│       │       ├── cine_lax_info.csv
│       │       ├── cine_lax_ks.mat
│       │       ├── cine_sax_calib.mat
│       │       ├── cine_sax_info.csv
│       │       ├── cine_sax_ks.mat
│       │       ├── t1map_calib.mat
│       │       ├── t1map_info.csv
│       │       ├── t1map_ks.mat
│       │       ├── t2map_calib.mat
│       │       ├── t2map_info.csv
│       │       └── t2map_ks.mat
│       ├── P107
│       │       ├── cine_lax_calib.mat
│       │       ├── cine_lax_info.csv
│       │       ├── cine_lax_ks.mat
│       │       ├── cine_sax_calib.mat
│       │       ├── cine_sax_info.csv
│       │       ├── cine_sax_ks.mat
│       │       ├── t1map_calib.mat
│       │       ├── t1map_info.csv
```

```
│       │       ├── t1map_ks.mat
│       │       ├── t2map_calib.mat
│       │       ├── t2map_info.csv
│       │       └── t2map_ks.mat
│       ├── P108
│       │       ├── cine_lax_calib.mat
│       │       ├── cine_lax_info.csv
│       │       ├── cine_lax_ks.mat
│       │       ├── cine_sax_calib.mat
│       │       ├── cine_sax_info.csv
│       │       ├── cine_sax_ks.mat
│       │       ├── t1map_calib.mat
│       │       ├── t1map_info.csv
│       │       ├── t1map_ks.mat
│       │       ├── t2map_calib.mat
│       │       ├── t2map_info.csv
│       │       └── t2map_ks.mat
│       ├── P109
│       │       ├── cine_lax_calib.mat
│       │       ├── cine_lax_info.csv
│       │       ├── cine_lax_ks.mat
│       │       ├── cine_sax_calib.mat
│       │       ├── cine_sax_info.csv
│       │       ├── cine_sax_ks.mat
│       │       ├── t1map_calib.mat
│       │       ├── t1map_info.csv
│       │       ├── t1map_ks.mat
│       │       ├── t2map_calib.mat
│       │       ├── t2map_info.csv
│       │       └── t2map_ks.mat
│       ├── P110
│       │       ├── cine_lax_calib.mat
│       │       ├── cine_lax_info.csv
│       │       ├── cine_lax_ks.mat
│       │       ├── cine_sax_calib.mat
│       │       ├── cine_sax_info.csv
│       │       ├── cine_sax_ks.mat
│       │       ├── t1map_calib.mat
│       │       ├── t1map_info.csv
│       │       ├── t1map_ks.mat
│       │       ├── t2map_calib.mat
│       │       ├── t2map_info.csv
│       │       └── t2map_ks.mat
│       ├── P111
│       │       ├── cine_lax_calib.mat
│       │       ├── cine_lax_info.csv
│       │       ├── cine_lax_ks.mat
│       │       ├── cine_sax_calib.mat
│       │       ├── cine_sax_info.csv
│       │       ├── cine_sax_ks.mat
│       │       ├── t1map_calib.mat
│       │       ├── t1map_info.csv
│       │       ├── t1map_ks.mat
│       │       ├── t2map_calib.mat
```

```
│       │       ├── t2map_info.csv
│       │       └── t2map_ks.mat
│       ├── P112
│       │       ├── cine_lax_calib.mat
│       │       ├── cine_lax_info.csv
│       │       ├── cine_lax_ks.mat
│       │       ├── cine_sax_calib.mat
│       │       ├── cine_sax_info.csv
│       │       ├── cine_sax_ks.mat
│       │       ├── t1map_calib.mat
│       │       ├── t1map_info.csv
│       │       ├── t1map_ks.mat
│       │       ├── t2map_calib.mat
│       │       ├── t2map_info.csv
│       │       └── t2map_ks.mat
│       ├── P113
│       │       ├── cine_lax_calib.mat
│       │       ├── cine_lax_info.csv
│       │       ├── cine_lax_ks.mat
│       │       ├── cine_sax_calib.mat
│       │       ├── cine_sax_info.csv
│       │       ├── cine_sax_ks.mat
│       │       ├── t1map_calib.mat
│       │       ├── t1map_info.csv
│       │       ├── t1map_ks.mat
│       │       ├── t2map_calib.mat
│       │       ├── t2map_info.csv
│       │       └── t2map_ks.mat
│       ├── P114
│       │       ├── cine_lax_calib.mat
│       │       ├── cine_lax_info.csv
│       │       ├── cine_lax_ks.mat
│       │       ├── cine_sax_calib.mat
│       │       ├── cine_sax_info.csv
│       │       ├── cine_sax_ks.mat
│       │       ├── t1map_calib.mat
│       │       ├── t1map_info.csv
│       │       ├── t1map_ks.mat
│       │       ├── t2map_calib.mat
│       │       ├── t2map_info.csv
│       │       └── t2map_ks.mat
│       ├── P115
│       │       ├── cine_lax_calib.mat
│       │       ├── cine_lax_info.csv
│       │       ├── cine_lax_ks.mat
│       │       ├── cine_sax_calib.mat
│       │       ├── cine_sax_info.csv
│       │       ├── cine_sax_ks.mat
│       │       ├── t1map_calib.mat
│       │       ├── t1map_info.csv
│       │       ├── t1map_ks.mat
│       │       ├── t2map_calib.mat
│       │       ├── t2map_info.csv
│       │       └── t2map_ks.mat
```

```
│       ├── P116
│       │   ├── cine_lax_calib.mat
│       │   ├── cine_lax_info.csv
│       │   ├── cine_lax_ks.mat
│       │   ├── cine_sax_calib.mat
│       │   ├── cine_sax_info.csv
│       │   ├── cine_sax_ks.mat
│       │   ├── t1map_calib.mat
│       │   ├── t1map_info.csv
│       │   ├── t1map_ks.mat
│       │   ├── t2map_calib.mat
│       │   ├── t2map_info.csv
│       │   └── t2map_ks.mat
│       ├── P117
│       │   ├── cine_lax_calib.mat
│       │   ├── cine_lax_info.csv
│       │   ├── cine_lax_ks.mat
│       │   ├── cine_sax_calib.mat
│       │   ├── cine_sax_info.csv
│       │   ├── cine_sax_ks.mat
│       │   ├── t1map_calib.mat
│       │   ├── t1map_info.csv
│       │   ├── t1map_ks.mat
│       │   ├── t2map_calib.mat
│       │   ├── t2map_info.csv
│       │   └── t2map_ks.mat
│       ├── P118
│       │   ├── cine_lax_calib.mat
│       │   ├── cine_lax_info.csv
│       │   ├── cine_lax_ks.mat
│       │   ├── cine_sax_calib.mat
│       │   ├── cine_sax_info.csv
│       │   ├── cine_sax_ks.mat
│       │   ├── t1map_calib.mat
│       │   ├── t1map_info.csv
│       │   ├── t1map_ks.mat
│       │   ├── t2map_calib.mat
│       │   ├── t2map_info.csv
│       │   └── t2map_ks.mat
│       ├── P119
│       │   ├── cine_lax_calib.mat
│       │   ├── cine_lax_info.csv
│       │   ├── cine_lax_ks.mat
│       │   ├── cine_sax_calib.mat
│       │   ├── cine_sax_info.csv
│       │   ├── cine_sax_ks.mat
│       │   ├── t1map_calib.mat
│       │   ├── t1map_info.csv
│       │   ├── t1map_ks.mat
│       │   ├── t2map_calib.mat
│       │   ├── t2map_info.csv
│       │   └── t2map_ks.mat
│       └── P120
│           ├── cine_lax_calib.mat
```

```
│       ├── cine_lax_info.csv
│       ├── cine_lax_ks.mat
│       ├── cine_sax_calib.mat
│       ├── cine_sax_info.csv
│       ├── cine_sax_ks.mat
│       ├── t1map_calib.mat
│       ├── t1map_info.csv
│       ├── t1map_ks.mat
│       ├── t2map_calib.mat
│       ├── t2map_info.csv
│       └── t2map_ks.mat
├── ValidationSet
│   ├── P001
│   │   ├── cine_lax_calib.mat
│   │   ├── cine_lax_info.csv
│   │   ├── cine_lax_ks.mat
│   │   ├── cine_sax_calib.mat
│   │   ├── cine_sax_info.csv
│   │   ├── cine_sax_ks.mat
│   │   ├── t1map_calib.mat
│   │   ├── t1map_info.csv
│   │   ├── t1map_ks.mat
│   │   ├── t2map_calib.mat
│   │   ├── t2map_info.csv
│   │   └── t2map_ks.mat
│   ├── P002
│   │   ├── cine_lax_calib.mat
│   │   ├── cine_lax_info.csv
│   │   ├── cine_lax_ks.mat
│   │   ├── cine_sax_calib.mat
│   │   ├── cine_sax_info.csv
│   │   ├── cine_sax_ks.mat
│   │   ├── t1map_calib.mat
│   │   ├── t1map_info.csv
│   │   ├── t1map_ks.mat
│   │   ├── t2map_calib.mat
│   │   ├── t2map_info.csv
│   │   └── t2map_ks.mat
│   ├── P003
│   │   ├── cine_lax_calib.mat
│   │   ├── cine_lax_info.csv
│   │   ├── cine_lax_ks.mat
│   │   ├── cine_sax_calib.mat
│   │   ├── cine_sax_info.csv
│   │   ├── cine_sax_ks.mat
│   │   ├── t1map_calib.mat
│   │   ├── t1map_info.csv
│   │   ├── t1map_ks.mat
│   │   ├── t2map_calib.mat
│   │   ├── t2map_info.csv
│   │   └── t2map_ks.mat
│   ├── P004
│   │   ├── cine_lax_calib.mat
│   │   ├── cine_lax_info.csv
```

```
│       │   ├── cine_lax_ks.mat
│       │   ├── cine_sax_calib.mat
│       │   ├── cine_sax_info.csv
│       │   ├── cine_sax_ks.mat
│       │   ├── t1map_calib.mat
│       │   ├── t1map_info.csv
│       │   ├── t1map_ks.mat
│       │   ├── t2map_calib.mat
│       │   ├── t2map_info.csv
│       │   └── t2map_ks.mat
│       ├── P005
│       │   ├── cine_lax_calib.mat
│       │   ├── cine_lax_info.csv
│       │   ├── cine_lax_ks.mat
│       │   ├── cine_sax_calib.mat
│       │   ├── cine_sax_info.csv
│       │   ├── cine_sax_ks.mat
│       │   ├── t1map_calib.mat
│       │   ├── t1map_info.csv
│       │   ├── t1map_ks.mat
│       │   ├── t2map_calib.mat
│       │   ├── t2map_info.csv
│       │   └── t2map_ks.mat
│       ├── P006
│       │   ├── cine_lax_calib.mat
│       │   ├── cine_lax_info.csv
│       │   ├── cine_lax_ks.mat
│       │   ├── cine_sax_calib.mat
│       │   ├── cine_sax_info.csv
│       │   ├── cine_sax_ks.mat
│       │   ├── t1map_calib.mat
│       │   ├── t1map_info.csv
│       │   ├── t1map_ks.mat
│       │   ├── t2map_calib.mat
│       │   ├── t2map_info.csv
│       │   └── t2map_ks.mat
│       ├── P007
│       │   ├── cine_lax_calib.mat
│       │   ├── cine_lax_info.csv
│       │   ├── cine_lax_ks.mat
│       │   ├── cine_sax_calib.mat
│       │   ├── cine_sax_info.csv
│       │   ├── cine_sax_ks.mat
│       │   ├── t1map_calib.mat
│       │   ├── t1map_info.csv
│       │   ├── t1map_ks.mat
│       │   ├── t2map_calib.mat
│       │   ├── t2map_info.csv
│       │   └── t2map_ks.mat
│       ├── P008
│       │   ├── cine_lax_calib.mat
│       │   ├── cine_lax_info.csv
│       │   ├── cine_lax_ks.mat
│       │   ├── cine_sax_calib.mat
```

```
│       │       ├── cine_sax_info.csv
│       │       ├── cine_sax_ks.mat
│       │       ├── t1map_calib.mat
│       │       ├── t1map_info.csv
│       │       ├── t1map_ks.mat
│       │       ├── t2map_calib.mat
│       │       ├── t2map_info.csv
│       │       └── t2map_ks.mat
│       ├── P009
│       │       ├── cine_lax_calib.mat
│       │       ├── cine_lax_info.csv
│       │       ├── cine_lax_ks.mat
│       │       ├── cine_sax_calib.mat
│       │       ├── cine_sax_info.csv
│       │       ├── cine_sax_ks.mat
│       │       ├── t1map_calib.mat
│       │       ├── t1map_info.csv
│       │       ├── t1map_ks.mat
│       │       ├── t2map_calib.mat
│       │       ├── t2map_info.csv
│       │       └── t2map_ks.mat
│       ├── P010
│       │       ├── cine_lax_calib.mat
│       │       ├── cine_lax_info.csv
│       │       ├── cine_lax_ks.mat
│       │       ├── cine_sax_calib.mat
│       │       ├── cine_sax_info.csv
│       │       ├── cine_sax_ks.mat
│       │       ├── t1map_calib.mat
│       │       ├── t1map_info.csv
│       │       ├── t1map_ks.mat
│       │       ├── t2map_calib.mat
│       │       ├── t2map_info.csv
│       │       └── t2map_ks.mat
│       ├── P011
│       │       ├── cine_lax_calib.mat
│       │       ├── cine_lax_info.csv
│       │       ├── cine_lax_ks.mat
│       │       ├── cine_sax_calib.mat
│       │       ├── cine_sax_info.csv
│       │       ├── cine_sax_ks.mat
│       │       ├── t1map_calib.mat
│       │       ├── t1map_info.csv
│       │       ├── t1map_ks.mat
│       │       ├── t2map_calib.mat
│       │       ├── t2map_info.csv
│       │       └── t2map_ks.mat
│       ├── P012
│       │       ├── cine_lax_calib.mat
│       │       ├── cine_lax_info.csv
│       │       ├── cine_lax_ks.mat
│       │       ├── cine_sax_calib.mat
│       │       ├── cine_sax_info.csv
│       │       ├── cine_sax_ks.mat
```

```
│       │       ├── t1map_calib.mat
│       │       ├── t1map_info.csv
│       │       ├── t1map_ks.mat
│       │       ├── t2map_calib.mat
│       │       ├── t2map_info.csv
│       │       └── t2map_ks.mat
│       ├── P013
│       │       ├── cine_lax_calib.mat
│       │       ├── cine_lax_info.csv
│       │       ├── cine_lax_ks.mat
│       │       ├── cine_sax_calib.mat
│       │       ├── cine_sax_info.csv
│       │       ├── cine_sax_ks.mat
│       │       ├── t1map_calib.mat
│       │       ├── t1map_info.csv
│       │       ├── t1map_ks.mat
│       │       ├── t2map_calib.mat
│       │       ├── t2map_info.csv
│       │       └── t2map_ks.mat
│       ├── P014
│       │       ├── cine_lax_calib.mat
│       │       ├── cine_lax_info.csv
│       │       ├── cine_lax_ks.mat
│       │       ├── cine_sax_calib.mat
│       │       ├── cine_sax_info.csv
│       │       ├── cine_sax_ks.mat
│       │       ├── t1map_calib.mat
│       │       ├── t1map_info.csv
│       │       ├── t1map_ks.mat
│       │       ├── t2map_calib.mat
│       │       ├── t2map_info.csv
│       │       └── t2map_ks.mat
│       ├── P015
│       │       ├── cine_lax_calib.mat
│       │       ├── cine_lax_info.csv
│       │       ├── cine_lax_ks.mat
│       │       ├── cine_sax_calib.mat
│       │       ├── cine_sax_info.csv
│       │       ├── cine_sax_ks.mat
│       │       ├── t1map_calib.mat
│       │       ├── t1map_info.csv
│       │       ├── t1map_ks.mat
│       │       ├── t2map_calib.mat
│       │       ├── t2map_info.csv
│       │       └── t2map_ks.mat
│       ├── P016
│       │       ├── cine_lax_calib.mat
│       │       ├── cine_lax_info.csv
│       │       ├── cine_lax_ks.mat
│       │       ├── cine_sax_calib.mat
│       │       ├── cine_sax_info.csv
│       │       ├── cine_sax_ks.mat
│       │       ├── t1map_calib.mat
│       │       ├── t1map_info.csv
```

```
│       │       ├── t1map_ks.mat
│       │       ├── t2map_calib.mat
│       │       ├── t2map_info.csv
│       │       └── t2map_ks.mat
│       ├── P017
│       │       ├── cine_lax_calib.mat
│       │       ├── cine_lax_info.csv
│       │       ├── cine_lax_ks.mat
│       │       ├── cine_sax_calib.mat
│       │       ├── cine_sax_info.csv
│       │       ├── cine_sax_ks.mat
│       │       ├── t1map_calib.mat
│       │       ├── t1map_info.csv
│       │       ├── t1map_ks.mat
│       │       ├── t2map_calib.mat
│       │       ├── t2map_info.csv
│       │       └── t2map_ks.mat
│       ├── P018
│       │       ├── cine_lax_calib.mat
│       │       ├── cine_lax_info.csv
│       │       ├── cine_lax_ks.mat
│       │       ├── cine_sax_calib.mat
│       │       ├── cine_sax_info.csv
│       │       ├── cine_sax_ks.mat
│       │       ├── t1map_calib.mat
│       │       ├── t1map_info.csv
│       │       ├── t1map_ks.mat
│       │       ├── t2map_calib.mat
│       │       ├── t2map_info.csv
│       │       └── t2map_ks.mat
│       ├── P019
│       │       ├── cine_lax_calib.mat
│       │       ├── cine_lax_info.csv
│       │       ├── cine_lax_ks.mat
│       │       ├── cine_sax_calib.mat
│       │       ├── cine_sax_info.csv
│       │       ├── cine_sax_ks.mat
│       │       ├── t1map_calib.mat
│       │       ├── t1map_info.csv
│       │       ├── t1map_ks.mat
│       │       ├── t2map_calib.mat
│       │       ├── t2map_info.csv
│       │       └── t2map_ks.mat
│       ├── P020
│       │       ├── cine_lax_calib.mat
│       │       ├── cine_lax_info.csv
│       │       ├── cine_lax_ks.mat
│       │       ├── cine_sax_calib.mat
│       │       ├── cine_sax_info.csv
│       │       ├── cine_sax_ks.mat
│       │       ├── t1map_calib.mat
│       │       ├── t1map_info.csv
│       │       ├── t1map_ks.mat
│       │       ├── t2map_calib.mat
```

```
│       │       ├── t2map_info.csv
│       │       └── t2map_ks.mat
│       ├── P021
│       │       ├── cine_lax_calib.mat
│       │       ├── cine_lax_info.csv
│       │       ├── cine_lax_ks.mat
│       │       ├── cine_sax_calib.mat
│       │       ├── cine_sax_info.csv
│       │       ├── cine_sax_ks.mat
│       │       ├── t1map_calib.mat
│       │       ├── t1map_info.csv
│       │       ├── t1map_ks.mat
│       │       ├── t2map_calib.mat
│       │       ├── t2map_info.csv
│       │       └── t2map_ks.mat
│       ├── P022
│       │       ├── cine_lax_calib.mat
│       │       ├── cine_lax_info.csv
│       │       ├── cine_lax_ks.mat
│       │       ├── cine_sax_calib.mat
│       │       ├── cine_sax_info.csv
│       │       ├── cine_sax_ks.mat
│       │       ├── t1map_calib.mat
│       │       ├── t1map_info.csv
│       │       ├── t1map_ks.mat
│       │       ├── t2map_calib.mat
│       │       ├── t2map_info.csv
│       │       └── t2map_ks.mat
│       ├── P023
│       │       ├── cine_lax_calib.mat
│       │       ├── cine_lax_info.csv
│       │       ├── cine_lax_ks.mat
│       │       ├── cine_sax_calib.mat
│       │       ├── cine_sax_info.csv
│       │       ├── cine_sax_ks.mat
│       │       ├── t1map_calib.mat
│       │       ├── t1map_info.csv
│       │       ├── t1map_ks.mat
│       │       ├── t2map_calib.mat
│       │       ├── t2map_info.csv
│       │       └── t2map_ks.mat
│       ├── P024
│       │       ├── cine_lax_calib.mat
│       │       ├── cine_lax_info.csv
│       │       ├── cine_lax_ks.mat
│       │       ├── cine_sax_calib.mat
│       │       ├── cine_sax_info.csv
│       │       ├── cine_sax_ks.mat
│       │       ├── t1map_calib.mat
│       │       ├── t1map_info.csv
│       │       ├── t1map_ks.mat
│       │       ├── t2map_calib.mat
│       │       ├── t2map_info.csv
│       │       └── t2map_ks.mat
```

```
│       ├── P025
│       │   ├── cine_lax_calib.mat
│       │   ├── cine_lax_info.csv
│       │   ├── cine_lax_ks.mat
│       │   ├── cine_sax_calib.mat
│       │   ├── cine_sax_info.csv
│       │   ├── cine_sax_ks.mat
│       │   ├── t1map_calib.mat
│       │   ├── t1map_info.csv
│       │   ├── t1map_ks.mat
│       │   ├── t2map_calib.mat
│       │   ├── t2map_info.csv
│       │   └── t2map_ks.mat
│       ├── P026
│       │   ├── cine_lax_calib.mat
│       │   ├── cine_lax_info.csv
│       │   ├── cine_lax_ks.mat
│       │   ├── cine_sax_calib.mat
│       │   ├── cine_sax_info.csv
│       │   ├── cine_sax_ks.mat
│       │   ├── t1map_calib.mat
│       │   ├── t1map_info.csv
│       │   ├── t1map_ks.mat
│       │   ├── t2map_calib.mat
│       │   ├── t2map_info.csv
│       │   └── t2map_ks.mat
│       ├── P027
│       │   ├── cine_lax_calib.mat
│       │   ├── cine_lax_info.csv
│       │   ├── cine_lax_ks.mat
│       │   ├── cine_sax_calib.mat
│       │   ├── cine_sax_info.csv
│       │   ├── cine_sax_ks.mat
│       │   ├── t1map_calib.mat
│       │   ├── t1map_info.csv
│       │   ├── t1map_ks.mat
│       │   ├── t2map_calib.mat
│       │   ├── t2map_info.csv
│       │   └── t2map_ks.mat
│       ├── P028
│       │   ├── cine_lax_calib.mat
│       │   ├── cine_lax_info.csv
│       │   ├── cine_lax_ks.mat
│       │   ├── cine_sax_calib.mat
│       │   ├── cine_sax_info.csv
│       │   ├── cine_sax_ks.mat
│       │   ├── t1map_calib.mat
│       │   ├── t1map_info.csv
│       │   ├── t1map_ks.mat
│       │   ├── t2map_calib.mat
│       │   ├── t2map_info.csv
│       │   └── t2map_ks.mat
│       ├── P029
│       │   ├── cine_lax_calib.mat
```

```
│       │       ├── cine_lax_info.csv
│       │       ├── cine_lax_ks.mat
│       │       ├── cine_sax_calib.mat
│       │       ├── cine_sax_info.csv
│       │       ├── cine_sax_ks.mat
│       │       ├── t1map_calib.mat
│       │       ├── t1map_info.csv
│       │       ├── t1map_ks.mat
│       │       ├── t2map_calib.mat
│       │       ├── t2map_info.csv
│       │       └── t2map_ks.mat
│       ├── P030
│       │       ├── cine_lax_calib.mat
│       │       ├── cine_lax_info.csv
│       │       ├── cine_lax_ks.mat
│       │       ├── cine_sax_calib.mat
│       │       ├── cine_sax_info.csv
│       │       ├── cine_sax_ks.mat
│       │       ├── t1map_calib.mat
│       │       ├── t1map_info.csv
│       │       ├── t1map_ks.mat
│       │       ├── t2map_calib.mat
│       │       ├── t2map_info.csv
│       │       └── t2map_ks.mat
│       ├── P031
│       │       ├── cine_lax_calib.mat
│       │       ├── cine_lax_info.csv
│       │       ├── cine_lax_ks.mat
│       │       ├── cine_sax_calib.mat
│       │       ├── cine_sax_info.csv
│       │       ├── cine_sax_ks.mat
│       │       ├── t1map_calib.mat
│       │       ├── t1map_info.csv
│       │       ├── t1map_ks.mat
│       │       ├── t2map_calib.mat
│       │       ├── t2map_info.csv
│       │       └── t2map_ks.mat
│       ├── P032
│       │       ├── cine_lax_calib.mat
│       │       ├── cine_lax_info.csv
│       │       ├── cine_lax_ks.mat
│       │       ├── cine_sax_calib.mat
│       │       ├── cine_sax_info.csv
│       │       ├── cine_sax_ks.mat
│       │       ├── t1map_calib.mat
│       │       ├── t1map_info.csv
│       │       ├── t1map_ks.mat
│       │       ├── t2map_calib.mat
│       │       ├── t2map_info.csv
│       │       └── t2map_ks.mat
│       ├── P033
│       │       ├── cine_lax_calib.mat
│       │       ├── cine_lax_info.csv
│       │       ├── cine_lax_ks.mat
```

```
│       │     ├── cine_sax_calib.mat
│       │     ├── cine_sax_info.csv
│       │     ├── cine_sax_ks.mat
│       │     ├── t1map_calib.mat
│       │     ├── t1map_info.csv
│       │     ├── t1map_ks.mat
│       │     ├── t2map_calib.mat
│       │     ├── t2map_info.csv
│       │     └── t2map_ks.mat
│       ├── P034
│       │     ├── cine_lax_calib.mat
│       │     ├── cine_lax_info.csv
│       │     ├── cine_lax_ks.mat
│       │     ├── cine_sax_calib.mat
│       │     ├── cine_sax_info.csv
│       │     ├── cine_sax_ks.mat
│       │     ├── t1map_calib.mat
│       │     ├── t1map_info.csv
│       │     ├── t1map_ks.mat
│       │     ├── t2map_calib.mat
│       │     ├── t2map_info.csv
│       │     └── t2map_ks.mat
│       ├── P035
│       │     ├── cine_lax_calib.mat
│       │     ├── cine_lax_info.csv
│       │     ├── cine_lax_ks.mat
│       │     ├── cine_sax_calib.mat
│       │     ├── cine_sax_info.csv
│       │     ├── cine_sax_ks.mat
│       │     ├── t1map_calib.mat
│       │     ├── t1map_info.csv
│       │     ├── t1map_ks.mat
│       │     ├── t2map_calib.mat
│       │     ├── t2map_info.csv
│       │     └── t2map_ks.mat
│       ├── P036
│       │     ├── cine_lax_calib.mat
│       │     ├── cine_lax_info.csv
│       │     ├── cine_lax_ks.mat
│       │     ├── cine_sax_calib.mat
│       │     ├── cine_sax_info.csv
│       │     ├── cine_sax_ks.mat
│       │     ├── t1map_calib.mat
│       │     ├── t1map_info.csv
│       │     ├── t1map_ks.mat
│       │     ├── t2map_calib.mat
│       │     ├── t2map_info.csv
│       │     └── t2map_ks.mat
│       ├── P037
│       │     ├── cine_lax_calib.mat
│       │     ├── cine_lax_info.csv
│       │     ├── cine_lax_ks.mat
│       │     ├── cine_sax_calib.mat
│       │     ├── cine_sax_info.csv
```

```
│       │       ├── cine_sax_ks.mat
│       │       ├── t1map_calib.mat
│       │       ├── t1map_info.csv
│       │       ├── t1map_ks.mat
│       │       ├── t2map_calib.mat
│       │       ├── t2map_info.csv
│       │       └── t2map_ks.mat
│       ├── P038
│       │       ├── cine_lax_calib.mat
│       │       ├── cine_lax_info.csv
│       │       ├── cine_lax_ks.mat
│       │       ├── cine_sax_calib.mat
│       │       ├── cine_sax_info.csv
│       │       ├── cine_sax_ks.mat
│       │       ├── t1map_calib.mat
│       │       ├── t1map_info.csv
│       │       ├── t1map_ks.mat
│       │       ├── t2map_calib.mat
│       │       ├── t2map_info.csv
│       │       └── t2map_ks.mat
│       ├── P039
│       │       ├── cine_lax_calib.mat
│       │       ├── cine_lax_info.csv
│       │       ├── cine_lax_ks.mat
│       │       ├── cine_sax_calib.mat
│       │       ├── cine_sax_info.csv
│       │       ├── cine_sax_ks.mat
│       │       ├── t1map_calib.mat
│       │       ├── t1map_info.csv
│       │       ├── t1map_ks.mat
│       │       ├── t2map_calib.mat
│       │       ├── t2map_info.csv
│       │       └── t2map_ks.mat
│       ├── P040
│       │       ├── cine_lax_calib.mat
│       │       ├── cine_lax_info.csv
│       │       ├── cine_lax_ks.mat
│       │       ├── cine_sax_calib.mat
│       │       ├── cine_sax_info.csv
│       │       ├── cine_sax_ks.mat
│       │       ├── t1map_calib.mat
│       │       ├── t1map_info.csv
│       │       ├── t1map_ks.mat
│       │       ├── t2map_calib.mat
│       │       ├── t2map_info.csv
│       │       └── t2map_ks.mat
│       ├── P041
│       │       ├── cine_lax_calib.mat
│       │       ├── cine_lax_info.csv
│       │       ├── cine_lax_ks.mat
│       │       ├── cine_sax_calib.mat
│       │       ├── cine_sax_info.csv
│       │       ├── cine_sax_ks.mat
│       │       ├── t1map_calib.mat
```

```
│       │       ├── t1map_info.csv
│       │       ├── t1map_ks.mat
│       │       ├── t2map_calib.mat
│       │       ├── t2map_info.csv
│       │       └── t2map_ks.mat
│       ├── P042
│       │       ├── cine_lax_calib.mat
│       │       ├── cine_lax_info.csv
│       │       ├── cine_lax_ks.mat
│       │       ├── cine_sax_calib.mat
│       │       ├── cine_sax_info.csv
│       │       ├── cine_sax_ks.mat
│       │       ├── t1map_calib.mat
│       │       ├── t1map_info.csv
│       │       ├── t1map_ks.mat
│       │       ├── t2map_calib.mat
│       │       ├── t2map_info.csv
│       │       └── t2map_ks.mat
│       ├── P043
│       │       ├── cine_lax_calib.mat
│       │       ├── cine_lax_info.csv
│       │       ├── cine_lax_ks.mat
│       │       ├── cine_sax_calib.mat
│       │       ├── cine_sax_info.csv
│       │       ├── cine_sax_ks.mat
│       │       ├── t1map_calib.mat
│       │       ├── t1map_info.csv
│       │       ├── t1map_ks.mat
│       │       ├── t2map_calib.mat
│       │       ├── t2map_info.csv
│       │       └── t2map_ks.mat
│       ├── P044
│       │       ├── cine_lax_calib.mat
│       │       ├── cine_lax_info.csv
│       │       ├── cine_lax_ks.mat
│       │       ├── cine_sax_calib.mat
│       │       ├── cine_sax_info.csv
│       │       ├── cine_sax_ks.mat
│       │       ├── t1map_calib.mat
│       │       ├── t1map_info.csv
│       │       ├── t1map_ks.mat
│       │       ├── t2map_calib.mat
│       │       ├── t2map_info.csv
│       │       └── t2map_ks.mat
│       ├── P045
│       │       ├── cine_lax_calib.mat
│       │       ├── cine_lax_info.csv
│       │       ├── cine_lax_ks.mat
│       │       ├── cine_sax_calib.mat
│       │       ├── cine_sax_info.csv
│       │       ├── cine_sax_ks.mat
│       │       ├── t1map_calib.mat
│       │       ├── t1map_info.csv
│       │       ├── t1map_ks.mat
```

```
│       │       ├── t2map_calib.mat
│       │       ├── t2map_info.csv
│       │       └── t2map_ks.mat
│       ├── P046
│       │       ├── cine_lax_calib.mat
│       │       ├── cine_lax_info.csv
│       │       ├── cine_lax_ks.mat
│       │       ├── cine_sax_calib.mat
│       │       ├── cine_sax_info.csv
│       │       ├── cine_sax_ks.mat
│       │       ├── t1map_calib.mat
│       │       ├── t1map_info.csv
│       │       ├── t1map_ks.mat
│       │       ├── t2map_calib.mat
│       │       ├── t2map_info.csv
│       │       └── t2map_ks.mat
│       ├── P047
│       │       ├── cine_lax_calib.mat
│       │       ├── cine_lax_info.csv
│       │       ├── cine_lax_ks.mat
│       │       ├── cine_sax_calib.mat
│       │       ├── cine_sax_info.csv
│       │       ├── cine_sax_ks.mat
│       │       ├── t1map_calib.mat
│       │       ├── t1map_info.csv
│       │       ├── t1map_ks.mat
│       │       ├── t2map_calib.mat
│       │       ├── t2map_info.csv
│       │       └── t2map_ks.mat
│       ├── P048
│       │       ├── cine_lax_calib.mat
│       │       ├── cine_lax_info.csv
│       │       ├── cine_lax_ks.mat
│       │       ├── cine_sax_calib.mat
│       │       ├── cine_sax_info.csv
│       │       ├── cine_sax_ks.mat
│       │       ├── t1map_calib.mat
│       │       ├── t1map_info.csv
│       │       ├── t1map_ks.mat
│       │       ├── t2map_calib.mat
│       │       ├── t2map_info.csv
│       │       └── t2map_ks.mat
│       ├── P049
│       │       ├── cine_lax_calib.mat
│       │       ├── cine_lax_info.csv
│       │       ├── cine_lax_ks.mat
│       │       ├── cine_sax_calib.mat
│       │       ├── cine_sax_info.csv
│       │       ├── cine_sax_ks.mat
│       │       ├── t1map_calib.mat
│       │       ├── t1map_info.csv
│       │       ├── t1map_ks.mat
│       │       ├── t2map_calib.mat
│       │       ├── t2map_info.csv
```

```
│       │   └── t2map_ks.mat
│       ├── P050
│       │   ├── cine_lax_calib.mat
│       │   ├── cine_lax_info.csv
│       │   ├── cine_lax_ks.mat
│       │   ├── cine_sax_calib.mat
│       │   ├── cine_sax_info.csv
│       │   ├── cine_sax_ks.mat
│       │   ├── t1map_calib.mat
│       │   ├── t1map_info.csv
│       │   ├── t1map_ks.mat
│       │   ├── t2map_calib.mat
│       │   ├── t2map_info.csv
│       │   └── t2map_ks.mat
│       ├── P051
│       │   ├── cine_lax_calib.mat
│       │   ├── cine_lax_info.csv
│       │   ├── cine_lax_ks.mat
│       │   ├── cine_sax_calib.mat
│       │   ├── cine_sax_info.csv
│       │   ├── cine_sax_ks.mat
│       │   ├── t1map_calib.mat
│       │   ├── t1map_info.csv
│       │   ├── t1map_ks.mat
│       │   ├── t2map_calib.mat
│       │   ├── t2map_info.csv
│       │   └── t2map_ks.mat
│       ├── P052
│       │   ├── cine_lax_calib.mat
│       │   ├── cine_lax_info.csv
│       │   ├── cine_lax_ks.mat
│       │   ├── cine_sax_calib.mat
│       │   ├── cine_sax_info.csv
│       │   ├── cine_sax_ks.mat
│       │   ├── t1map_calib.mat
│       │   ├── t1map_info.csv
│       │   ├── t1map_ks.mat
│       │   ├── t2map_calib.mat
│       │   ├── t2map_info.csv
│       │   └── t2map_ks.mat
│       ├── P053
│       │   ├── cine_lax_calib.mat
│       │   ├── cine_lax_info.csv
│       │   ├── cine_lax_ks.mat
│       │   ├── cine_sax_calib.mat
│       │   ├── cine_sax_info.csv
│       │   ├── cine_sax_ks.mat
│       │   ├── t1map_calib.mat
│       │   ├── t1map_info.csv
│       │   ├── t1map_ks.mat
│       │   ├── t2map_calib.mat
│       │   ├── t2map_info.csv
│       │   └── t2map_ks.mat
│       ├── P054
```

```
│       │       ├── cine_lax_calib.mat
│       │       ├── cine_lax_info.csv
│       │       ├── cine_lax_ks.mat
│       │       ├── cine_sax_calib.mat
│       │       ├── cine_sax_info.csv
│       │       ├── cine_sax_ks.mat
│       │       ├── t1map_calib.mat
│       │       ├── t1map_info.csv
│       │       ├── t1map_ks.mat
│       │       ├── t2map_calib.mat
│       │       ├── t2map_info.csv
│       │       └── t2map_ks.mat
│       ├── P055
│       │       ├── cine_lax_calib.mat
│       │       ├── cine_lax_info.csv
│       │       ├── cine_lax_ks.mat
│       │       ├── cine_sax_calib.mat
│       │       ├── cine_sax_info.csv
│       │       ├── cine_sax_ks.mat
│       │       ├── t1map_calib.mat
│       │       ├── t1map_info.csv
│       │       ├── t1map_ks.mat
│       │       ├── t2map_calib.mat
│       │       ├── t2map_info.csv
│       │       └── t2map_ks.mat
│       ├── P056
│       │       ├── cine_lax_calib.mat
│       │       ├── cine_lax_info.csv
│       │       ├── cine_lax_ks.mat
│       │       ├── cine_sax_calib.mat
│       │       ├── cine_sax_info.csv
│       │       ├── cine_sax_ks.mat
│       │       ├── t1map_calib.mat
│       │       ├── t1map_info.csv
│       │       ├── t1map_ks.mat
│       │       ├── t2map_calib.mat
│       │       ├── t2map_info.csv
│       │       └── t2map_ks.mat
│       ├── P057
│       │       ├── cine_lax_calib.mat
│       │       ├── cine_lax_info.csv
│       │       ├── cine_lax_ks.mat
│       │       ├── cine_sax_calib.mat
│       │       ├── cine_sax_info.csv
│       │       ├── cine_sax_ks.mat
│       │       ├── t1map_calib.mat
│       │       ├── t1map_info.csv
│       │       ├── t1map_ks.mat
│       │       ├── t2map_calib.mat
│       │       ├── t2map_info.csv
│       │       └── t2map_ks.mat
│       ├── P058
│       │       ├── cine_lax_calib.mat
│       │       ├── cine_lax_info.csv
```

```
│   │   ├── cine_lax_ks.mat
│   │   ├── cine_sax_calib.mat
│   │   ├── cine_sax_info.csv
│   │   ├── cine_sax_ks.mat
│   │   ├── t1map_calib.mat
│   │   ├── t1map_info.csv
│   │   ├── t1map_ks.mat
│   │   ├── t2map_calib.mat
│   │   ├── t2map_info.csv
│   │   └── t2map_ks.mat
│   ├── P059
│   │   ├── cine_lax_calib.mat
│   │   ├── cine_lax_info.csv
│   │   ├── cine_lax_ks.mat
│   │   ├── cine_sax_calib.mat
│   │   ├── cine_sax_info.csv
│   │   ├── cine_sax_ks.mat
│   │   ├── t1map_calib.mat
│   │   ├── t1map_info.csv
│   │   ├── t1map_ks.mat
│   │   ├── t2map_calib.mat
│   │   ├── t2map_info.csv
│   │   └── t2map_ks.mat
│   └── P060
│       ├── cine_lax_calib.mat
│       ├── cine_lax_info.csv
│       ├── cine_lax_ks.mat
│       ├── cine_sax_calib.mat
│       ├── cine_sax_info.csv
│       ├── cine_sax_ks.mat
│       ├── t1map_calib.mat
│       ├── t1map_info.csv
│       ├── t1map_ks.mat
│       ├── t2map_calib.mat
│       ├── t2map_info.csv
│       └── t2map_ks.mat
│
```
